# Analysis of *Candida albicans* Mutants Defective in the Cdk8 Module of Mediator Reveal Links between Metabolism and Biofilm Formation

**DOI:** 10.1371/journal.pgen.1004567

**Published:** 2014-10-02

**Authors:** Allia K. Lindsay, Diana K. Morales, Zhongle Liu, Nora Grahl, Anda Zhang, Sven D. Willger, Lawrence C. Myers, Deborah A. Hogan

**Affiliations:** 1 Department of Microbiology and Immunology, Geisel School of Medicine at Dartmouth, Hanover, New Hampshire, United States of America; 2 Department of Biochemistry, Geisel School of Medicine at Dartmouth, Hanover, New Hampshire, United States of America; University College Dublin, Ireland

## Abstract

*Candida albicans* biofilm formation is a key virulence trait that involves hyphal growth and adhesin expression. Pyocyanin (PYO), a phenazine secreted by *Pseudomonas aeruginosa*, inhibits both *C. albicans* biofilm formation and development of wrinkled colonies. Using a genetic screen, we identified two mutants, *ssn3*Δ/Δ and *ssn8*Δ/Δ, which continued to wrinkle in the presence of PYO. Ssn8 is a cyclin-like protein and Ssn3 is similar to cyclin-dependent kinases; both proteins are part of the heterotetrameric Cdk8 module that forms a complex with the transcriptional co-regulator, Mediator. Ssn3 kinase activity was also required for PYO sensitivity as a kinase dead mutant maintained a wrinkled colony morphology in the presence of PYO. Furthermore, similar phenotypes were observed in mutants lacking the other two components of the Cdk8 module—Srb8 and Srb9. Through metabolomics analyses and biochemical assays, we showed that a compromised Cdk8 module led to increases in glucose consumption, glycolysis-related transcripts, oxidative metabolism and ATP levels even in the presence of PYO. In the mutant, inhibition of respiration to levels comparable to the PYO-treated wild type inhibited wrinkled colony development. Several lines of evidence suggest that PYO does not act through Cdk8. Lastly, the *ssn3* mutant was a hyperbiofilm former, and maintained higher biofilm formation in the presence of PYO than the wild type. Together these data provide novel insights into the role of the Cdk8 module of Mediator in regulation of *C. albicans* physiology and the links between respiratory activity and both wrinkled colony and biofilm development.

## Author Summary

*Candida albicans* is currently one of the most common causes of nosocomial infections, and causes diseases ranging from oral thrush to life-threatening systemic infections. *C. albicans* readily forms biofilms on implanted devices, such as catheters and dentures, and biofilms are associated with increased risk of systemic infections and resistance to antifungals. We previously showed that biofilm formation positively correlates with oxidative metabolism. Here, we show that respiration, wrinkled colony formation, biofilm formation, and resistance to the inhibitory effects of pyocyanin were increased when the Cdk8 module of Mediator, a co-regulator of transcription, was compromised. Cdk8 module mutants all exhibited differences in basal metabolism that were indicative of increased metabolic activity. Furthermore, using these strains, we showed a direct correlation between the inhibition of respiratory activity, in the absence of growth defects, and inhibition of biofilm formation, supporting a model in which *C. albicans* metabolism and morphology are linked. The human Cdk8 module is currently being investigated as a target for cancer chemotherapy, and thus an understanding of the consequences or potential benefits of inhibiting this module is required.

## Introduction

*Candida albicans* is a fungus that can switch from growth as a natural human commensal to a pathogen and, if host defenses are compromised, consequently cause life-threatening systemic infections [1]–[6]. This organism readily forms biofilms, which are complex communities composed of various cellular morphologies that are held together by the production of adhesins and extracellular matrix polymeric substances that are mainly composed of polysaccharides [7]. Morphological plasticity is a key requirement for biofilm formation [8], as this process involves initial attachment to a surface by yeast cells which germinate to form a matrix-encased hyphal network [9]. As a natural member of the human microbiota, *C. albicans* readily encounters indwelling biomedical devices such as intravascular catheters that can act as biofilm substrates [10], [11]. Consequently, *C. albicans* is currently highly ranked among the fungi most commonly isolated from catheter-based biofilm infections [12], [13]. In addition, biofilm formation by *C. albicans* confers increased resistance to antifungal agents [14]–[18]. Given biofilms play a key role in *C. albicans* disease, it is important to understand the physiological pathways that can promote or prevent biofilm development.

*C. albicans* growth as wrinkled colonies also requires hypha formation and the production of adhesins [19]–[23]. Formation of these wrinkled communities by *C. albicans* strains has been found to facilitate increased access to oxygen and may thus serve as a means to maintain redox balance [24], [25]. Consistent with this model, low oxygen conditions stimulate filamentation and wrinkled colony formation in various *Candida* species [7], [26], [27]. In contrast, anoxic environments reportedly inhibit biofilm formation while still permitting hyphal growth [28], [29]. Interestingly, Watanabe and colleagues described that the induction of hyphal growth is positively regulated by the respiratory chain, due to their observation that disruption of electron flow between complex II and Coenzyme Q by treatment with thenoyltrifluoroacetone drastically repressed filamentation [30]. Supporting their observation, we recently reported that pyocyanin (PYO), a redox-active phenazine secreted by *Pseudomonas aeruginosa*, and related compounds (e.g. methylene blue) reduce respiratory activity and inhibit formation of wrinkled colonies and biofilms [25]. These findings suggest a link between respiration and biofilm formation in *C. albicans*.

**Figure 1:**
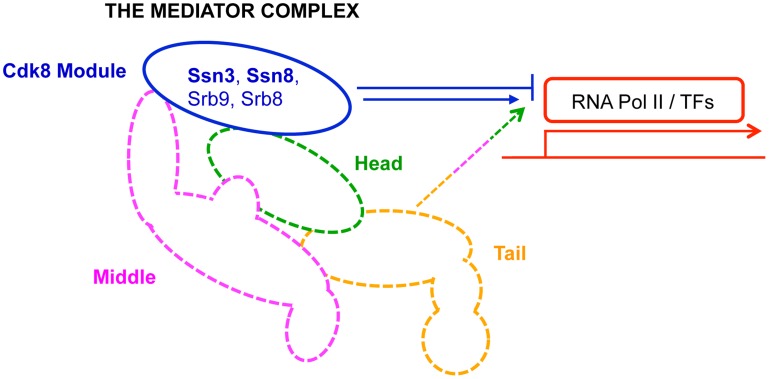
Primary role of Mediator modules in transcription. The Mediator complex is composed of a Core module (broken lines) and a Cdk8 module (solid blue line). The Core module is sub-divided into a head (green), middle (pink) and tail (orange) domain; each domain is composed of multiple subunits, and is primarily involved in activation of transcription. In contrast, the Cdk8 module (blue)—composed of Ssn3, Ssn8, Srb8 and Srb9—is primarily implicated in transcriptional repression. RNA Pol II: RNA polymerase II; TFs: transcription factors.

To gain further insight into the link between PYO-mediated inhibition of respiration and the inhibition of biofilm formation, we screened mutant collections to identify strains that continued to form wrinkled colonies in the presence of non-toxic concentrations of PYO. This genetic approach revealed that the absence of Ssn3 or Ssn8 increased resistance to PYO-induced repression of hyphal growth and colony wrinkling. Ssn3 and Ssn8 are components of the Cdk8 module of Mediator, which is a eukaryote-specific multi-protein complex [31] that serves as a bridge between regulatory proteins and RNA polymerase (Figure 1) [32], [33]. Mediator is an integral component of transcriptional regulation, and this process may be a key way by which cells can modulate the transition to biofilm or wrinkled colony formation. The heterotetrameric Cdk8 module of Mediator is primarily involved in transcriptional repression [34], [35] and studies in other fungi such as *Saccharomyces cerevisiae* and *Schizosaccharomyces pombe* have shown that this module regulates hundreds of genes, some of which have been linked to carbon source utilization, stress responses and adhesin expression [36]–[41]. We found that disruption of Cdk8 module activity, but not non-essential regulatory subunits of the Core module of Mediator, resulted in increased glucose uptake, metabolic activity and ATP production. These data suggested that increased respiratory activity conferred resistance to the effects of PYO. Our work indicated a tight link between metabolic activity and the formation of wrinkled colonies; it also showed that the stability of Ssn3 and Ssn8 were unaffected by PYO. Analysis of the metabolite profiles in the wild type and *ssn3* mutant grown in the absence and presence of PYO was performed; these data provided insight into both additional roles of Ssn3 and the effects of PYO on *C. albicans*. Taken together, these results increase our understanding of the role played by the Cdk8 module in the regulation of metabolism and morphological plasticity, the links between these two processes and the mechanism by which phenazines, such as PYO, affect the biology of *C. albicans*.

## Results

### Genetic screen of *C. albicans* mutants revealed that *ssn3* and *ssn8* mutants have increased resistance to the inhibitory effect of PYO on wrinkling

**Figure 2:**
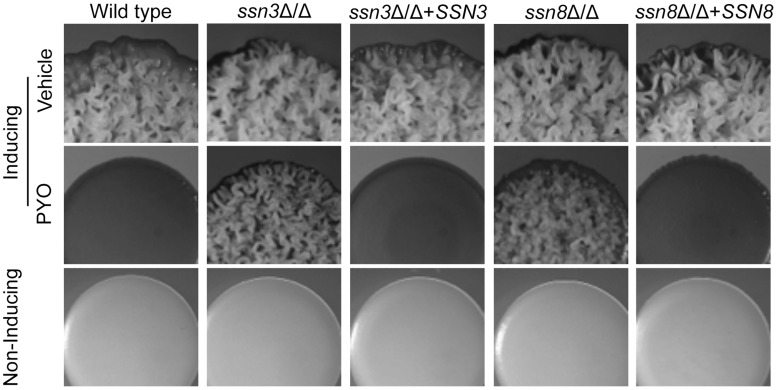
Genetic screen revealed that *ssn3* and *ssn8* mutants maintain wrinkling in the presence of PYO. Spot-inoculated colonies of wild type (SC5314), *ssn3*Δ/Δs, *ssn3*Δ/Δ+*SSN3* (complemented mutant), *ssn8*Δ/Δ and *ssn8*Δ/Δ+*SSN8* (complemented mutant) were grown on YNBAG_10_N-agar at 37°C (wrinkling-inducing conditions) in the presence of vehicle or 20 µM PYO. Strains were also grown on YNBG_100_-agar at 30°C (yeast growth conditions). Colonies were imaged with a dissecting stereoscope after 48 h of growth.

We previously demonstrated that PYO inhibits both biofilm formation on plastic and wrinkled colony formation [25]. Using the wrinkled colony phenotype that develops in *C. albicans* colonies when they are incubated at 37°C on medium with GlcNAc, we performed a genetic screen for mutants that were resistant to the repressive effects of PYO. In a screen of approximately 1,500 strains from homozygous knockout collections [42]–[46], only thirteen were found to wrinkle under inducing conditions in the presence of 20 µM PYO (Table S1). Eleven of the thirteen mutants also wrinkled under non-inducing conditions in which colonies formed by wild-type strains are smooth and contain yeast cells, and these were excluded from subsequent studies due to the lack of specific “phenazine-resistance” phenotype. The remaining two mutants, *ssn3*Δ/Δ and *ssn8*Δ/Δ, formed colonies similar to those of the wild type on non-inducing and inducing media without PYO, but formed wrinkled colonies in the presence of PYO. The PYO-resistance phenotype persisted when the mutants were restored to prototrophy using the vector pDDB78, and complementation with their respective native alleles restored sensitivity to PYO (Figure 2). Similar to the wild type strain, both mutants and their complemented derivatives formed smooth colonies under non-inducing conditions (Figure 2). Analysis of the cellular morphology showed that wrinkled colonies contained a mixture of hyphae and yeast on medium with vehicle [25]. PYO caused the wild type to grow as a smooth colony composed exclusively of yeast, while *ssn3* and *ssn8* mutant colonies contained both yeast and hyphal cells, as is characteristic of wrinkled colonies.

### Loss of Ssn3 kinase activity confers resistance to PYO

**Figure 3:**
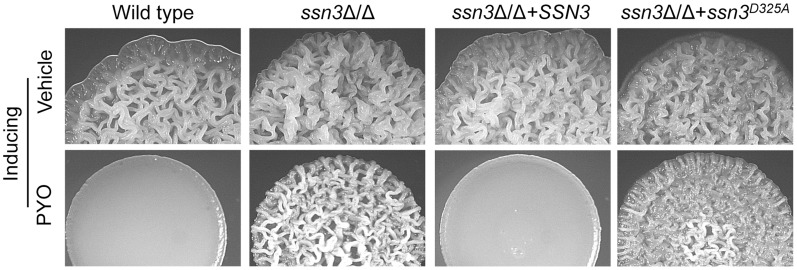
The Ssn3-Ssn8 cyclin kinase-cyclin pair influences sensitivity to PYO via a phosphorylation event. Colonies of wild type (SC5314), *ssn3*Δ/Δ, *ssn3*Δ/Δ+*SSN3* and the *ssn3*Δ/Δ+*ssn3^D325^* kinase dead mutant were grown on YNBAG_10_N-agar with vehicle or 20 µM PYO at 37°C for 48 h and then imaged with a dissecting stereoscope.

Ssn3 and Ssn8 of *C. albicans* are orthologous to *S. cerevisiae* Srb10 and Srb11, which make up a cyclin-dependent kinase/cyclin pair. These proteins are components of the Cdk8 module of Mediator (Figure 1), which is an RNA polymerase II (RNAPII) co-regulator of gene expression [32], [33]. The Cdk8 module is best known for its repressive activities while the Core module of Mediator generally promotes transcription [32], [35], [47]–[49]. Ssn3 (Srb10) kinase activity participates in transcriptional regulation through phosphorylation of RNAPII [50]–[52] and phosphorylation of target transcription factors [53]–[58]. We therefore determined if loss of Ssn3 kinase activity led to a phenotype similar to the *ssn3*Δ/Δ mutant, in terms of resistance to PYO, using the previously published strain expressing the *ssn3^D325A^* allele that is predicted to encode a kinase-dead Ssn3 variant [56]. Given the strain expressing the kinase-dead variant maintained wrinkling in the presence of PYO, whereas the comparable strain expressing the native allele was still sensitive, we concluded that the loss of Ssn3 kinase activity was sufficient to promote *C. albicans* PYO-resistance (Figure 3).

### The stability of Ssn3 and Ssn8 is unaffected by treatment with PYO

**Figure 4:**
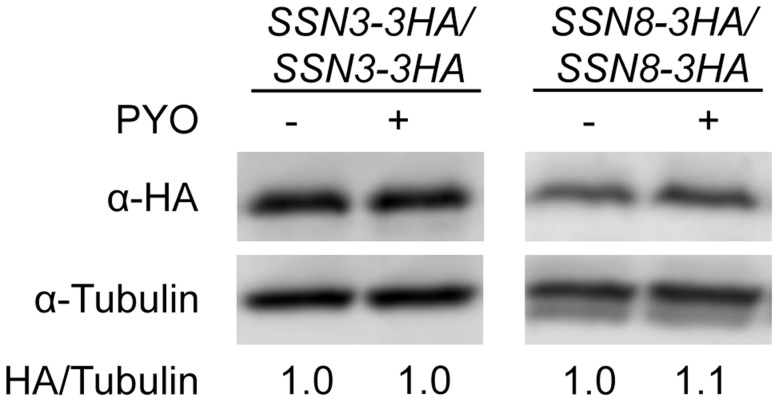
The stability of Ssn3 and Ssn8 is unaffected by PYO. Strains with both alleles of *SSN3* or *SSN8* encoding HA-tagged variants of the native protein were grown at 37°C in YNBG_10_NP and supplemented with uridine in the presence of vehicle or 20 µM PYO. Cultures were harvested for protein extraction at mid-exponential phase, when the OD_600 nm_ was ≈0.5. Calculated molecular weights for HA-tagged Ssn3, HA-tagged Ssn8 and tubulin are 73 kD, 54.4 kD and 50 kD and weight estimates, relative to the molecular weight standards, are 79 kD, 55 kD and 54 kD respectively.

In *S. cerevisiae*, changes in levels of the Ssn3 and Ssn8 orthologs have been shown to regulate transcriptional profiles [59]. Because the *ssn3* and *ssn8* mutants continued to wrinkle on medium with PYO, we sought to determine if PYO-induced repression of filamentation and wrinkled colony formation was mediated by increased levels of Ssn3, Ssn8 or both proteins. To test this model, we performed a Western blot analysis of strains bearing HA-tagged variants of either Ssn3 or Ssn8 grown to exponential phase in liquid medium, containing the filament inducer GlcNAc, in the presence and absence of PYO. While PYO inhibits filamentation under these growth conditions on both agar medium (Figure 2) and in liquid [25], it did not affect the levels of Ssn3-HA or Ssn8-HA (Figure 4) relative to the tubulin control, indicating that PYO-mediated repression of morphology was likely not due to increased levels of these proteins.

### Mutation of *SRB8* or *SRB9*, which also encode Cdk8 module proteins, increases resistance to the inhibitory effect of PYO on wrinkling

Having identified two mutants of the heterotetrameric Cdk8 module of Mediator as having increased resistance to PYO, we sought to determine if mutants lacking other components of the Cdk8 module—Srb8 and Srb9—also had increased resistance to PYO-mediated repression of wrinkling. We found that absence of either *SRB8* or *SRB9* also increased resistance to PYO (Figure S1A). Restoration of the *SRB9* gene complemented the PYO-resistance phenotype, though robust complementation was not achieved with a single allele of *SRB8* suggesting haploinsufficiency. Several reviews have described the Cdk8 module of Mediator acting primarily as a transcriptional repressor when in complex with the Core module, while the Core module without Cdk8 is largely involved in transcriptional activation (Figure 1) [32], [35], [47]–[49], [60]. Studies of *S. cerevisiae* suggest a high degree of diversity in the effects of non-essential Mediator subunits on transcription, however some Cdk8 module phenotypes are reportedly shared by the subunits of the Core module [38]. To determine if the PYO-resistance phenotype was also observed upon loss of other non-essential Mediator components, we tested the phenotypes for mutants lacking *MED1*, *MED3*, *MED5*, *MED9*, *MED16* and *MED20*. None of these Core module mutants maintained wrinkling in the presence of PYO (Figure S1B), though a slight level of resistance was observed in *med3*Δ/Δ. These data suggest that the PYO-resistance phenotype is specifically associated with defects in the Cdk8 module of Mediator.

In *S. cerevisiae*, the Cdk8 module can negatively regulate transcription factors that act in concert with Tup1 [36], [61]. In *C. albicans*, the loss of Tup1 or its co-regulator Nrg1 is sufficient to lead to constitutive filamentation and wrinkled colony formation [62], [63]. To determine if the phenotype of the Cdk8 module mutants could be attributed to lower levels of Nrg1 or other Tup1-co-regulators, we determined if *tup1*Δ/Δ and *nrg1*Δ/Δ strains were also resistant to the effects of PYO. This did not appear to be the case, as both mutants showed a marked reduction in wrinkling with PYO indicating that the Cdk8 module was not likely acting through Tup1 or Nrg1 in smooth colonies and that hyperwrinkling does not necessarily confer enhanced resistance to PYO (Figure S2).

### The hyperalkalinization phenotype of the *ssn3* mutant is not sufficient for resistance to PYO

**Figure 5:**
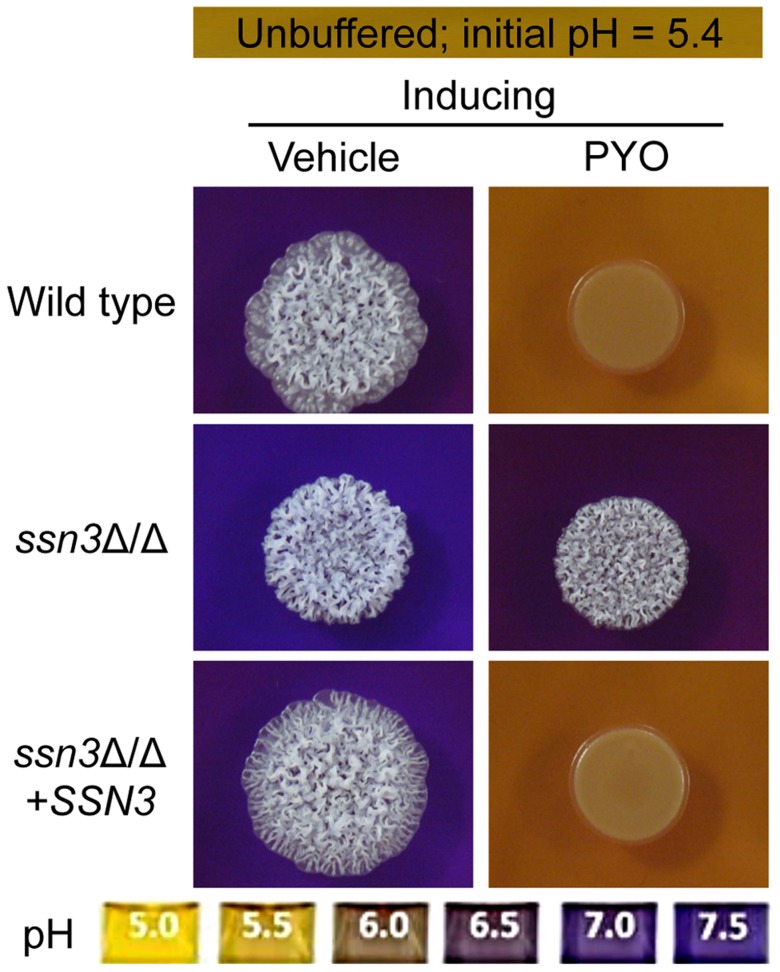
The *ssn3* mutant hyperalkalinizes the extracellular milieu even in the presence of PYO. Colonies of wild type (SC5314), *ssn3*Δ/Δ and *ssn3*Δ/Δ+*SSN3* were grown on YNBAG_10_N-agar containing 0.01% bromocresol purple in the presence of vehicle or 20 µM PYO for 48 h at 37°C and then imaged with a digital camera.

We previously reported that the inhibition of respiratory metabolism in *C. albicans* by PYO correlates with increased acetic acid production and a concomitant inability to alkalinize the extracellular milieu [25]. To determine if the Cdk8 module influenced the ability to alkalinize the medium, we added the pH indicator bromocresol purple to the medium to assess changes in extracellular pH over time. We initially focused on the *ssn3* mutant, which encodes the Cdk8-like kinase. Our results indicated that, in the absence of PYO, the *ssn3* mutant alkalinized the medium more quickly (Figure S3A) and to a greater extent (Figure 5 and S3B) than the wild-type controls. Furthermore, in contrast to the wild type [25] and complemented strain, the *ssn3* mutant alkalinized the medium even in the presence of PYO (Figure 5). We found similar phenotypes in the other three Cdk8 module mutants—*ssn8*, *srb8*, and *srb9* (Figure S3B); phenotypic similarity among different Cdk8 module mutants has been observed in other studies in other species [52], [64], [65].

Because pH is a strong modulator of morphology [66], [67], we sought to determine if the increased extracellular pH around *ssn3* mutant colonies contributed to the ability to filament in the presence of PYO. To test this idea, we cultured our strains of interest on inducing medium that was buffered to pH 7 in the presence or absence of PYO. Both strains formed wrinkled colonies on the buffered medium in the absence of PYO (Figure S4A), but while the filamentation of the wild type was still largely repressed by PYO on medium buffered to pH 7, the *ssn3* mutant continued to form wrinkled colonies (Figure S4A). Furthermore, on medium buffered to pH 7 containing glucose and GlcNAc without amino acids, the *ssn3*Δ/Δ strain wrinkled in both the presence and absence of PYO while the wild type was only wrinkled on PYO-free medium (Figure S4B). Thus, we concluded that differences in amino acid catabolism and the concomitant increases in the pH of the medium, were not sufficient to cause the persistent colony wrinkling of the *ssn3* mutant in the presence of PYO.

To determine if the changes in colony morphology induced by PYO were sufficient to cause differences in metabolism that led to increased acidification, wild type and mutant strains were grown under non-inducing (yeast growth) conditions in the presence and absence of PYO on medium containing a pH indicator. As under conditions that promote colony wrinkling, PYO prevented alkalinization of medium by the wild type, but not the *ssn3* mutant (Figure S4C) leading us to conclude that the mutant had fundamental differences in metabolism that were not dependent on changes in morphology. The alkalinization or other differences in metabolism in the *ssn3* mutant may, however, contribute to colony wrinkling as the addition of amino acids to the medium caused slight wrinkling in the *ssn3* mutant under yeast growth conditions (compare Figure S4C to Figure 2). Based on these findings, we concluded that the higher pH in *ssn3Δ/Δ* cultures was not sufficient to explain wrinkling in the presence of PYO, and that the effects of PYO on metabolism were independent of differences in colony and cellular morphology.

### Mutation of *SSN3* results in the perturbation of various metabolic pathways

We recently reported that the lower pH of the extracellular milieu in the presence of PYO is due to changes in fungal metabolism induced by this bacterial product [25]. The higher pH in mutant cultures, when compared to those of the wild type, prompted us to test the hypothesis that there was a difference in the basal metabolism of the *ssn3*Δ/Δ mutant compared to the wild type strain, and that these differences contributed to a change in PYO resistance. To gain insight into the role of the Cdk8 module in *C. albicans* metabolism, we performed a metabolomics analysis of the wild type, *ssn3*Δ/Δ, and *ssn3*Δ/Δ+*SSN3* strains. More than 250 metabolites were reported (Table S2), and we focused on those that were different between the *ssn3*Δ/Δ mutant and both the wild type and complemented strain, but were not different between the wild type and the complemented strain. The 221 metabolites that met this criterion were divided into eight metabolite categories: carbohydrate, amino acid, energy, nucleotide, peptide, lipid, cofactors & vitamins and xenobiotics (namely pyocyanin) (Table S2). Under our vehicle-treated conditions, the two categories with the largest number of compounds higher in the mutant were “carbohydrates” and “amino acids”. Interestingly, there were a large number of compounds at lower levels in the “amino acids” and “peptides” categories when the mutant was compared to the wild type and *SSN3* complemented strain (Figure S5). These categories included intermediates in both biosynthesis and catabolism of the canonical amino acids as well as several amino acid derivatives such as glutathione. Initially, we focused on the increased representation among the sugars and their derivatives; the patterns evident in amino acid and peptide categories are considered further in the Discussion.

### Mutation of *SSN3* results in increased glycolytic activity and glucose transport

Our metabolomics data suggested the intracellular levels of glucose and glucose-6-phoshate were higher in the mutant compared to the wild type, and led us to propose that the mutant had increased glycolytic activity (Table 1). Fructose-6-phosphate and 3-phosphoglycerate, two other glycolytic intermediates, showed similar increases in the mutant compared to the wild type and the *SSN3* complemented strain, but did not meet the criterion of statistical significance (p>0.05).

**Table 1:** Mutation of *SSN3* alters the catabolism of glucose.

Category	Metabolite	*ssn3* Δ/Δ	*SSN3*	*ssn3* Δ/Δ
		Wild type	Wild type	*SSN3*
*Glycolysis*	Glucose	**1.51**	1.07	**1.41**
	Glucose-6-phosphate	**1.65**	1.15	**1.43**
	Mannose-6-phosphate	**1.77**	1.07	**1.67**
	Fructose-6-phosphate	**1.57**	1.18	1.33
	3-phosphoglycerate	1.62	0.75	2.17
*PPP-related intermediates*	Threitol	**0.68**	0.97	**0.69**
	Erythronate	**0.70**	1.12	**0.63**
*PPP canonical intermediates*	Gluconate	**0.54**	1.32	**0.41**
	Glycerate	**0.72**	1.09	**0.66**
	Ribose	0.87	0.94	0.93
	Ribonate	0.97	0.87	1.12
	Ribose 5-phosphate	0.94	0.92	1.02
	Ribulose	0.76	0.86	0.88

Underlining represents a statistically significant difference (p≤0.05).

**Figure 6:**
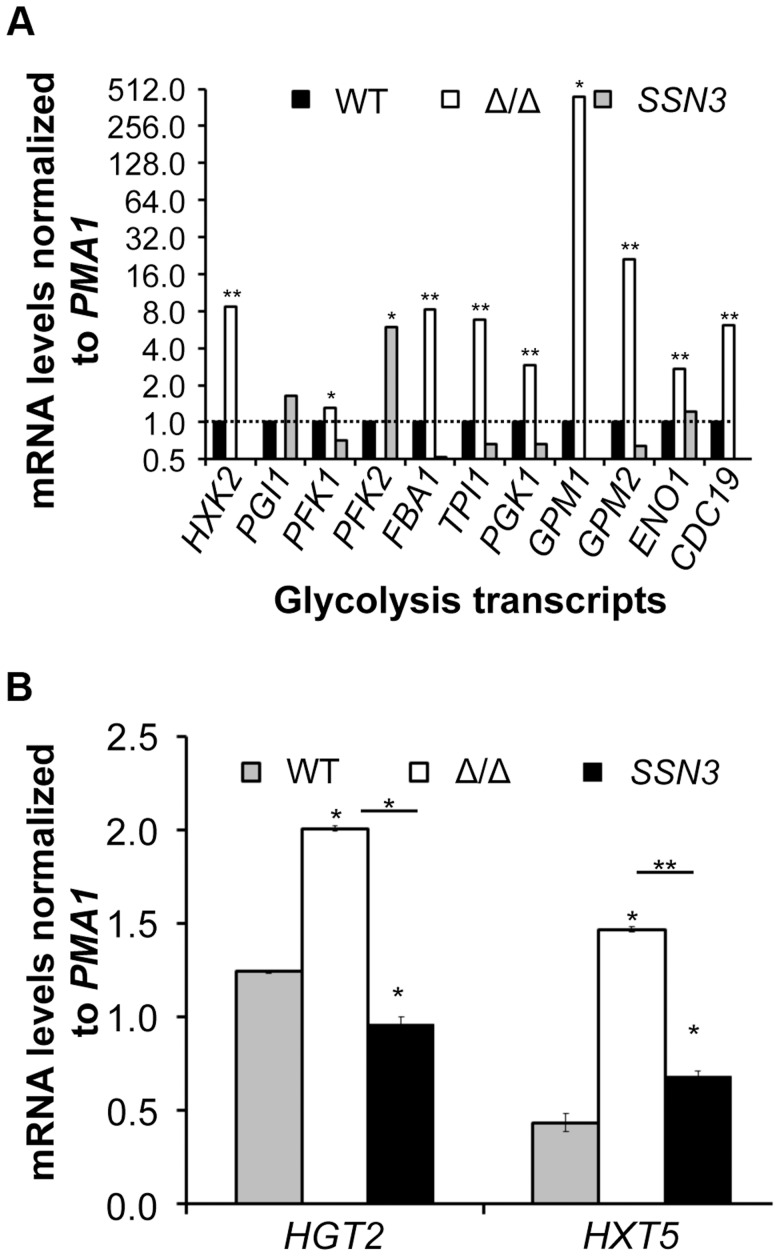
Loss of *SSN3* results in increased expression of glycolysis genes and glucose transporters. Wrinkled colonies of wild type (WT; SC5314), *ssn3*Δ/Δ (Δ/Δ) and *ssn3*Δ/Δ+*SSN3* (*SSN3*) were grown on YNBAG_10_N-agar for 24 h. Transcript levels of (**A**) *HXK2*, *PGI1*, *PFK1*, *PFK2*, *FBA1*, *TPI1*, *PGK1*, *GPM1*, *GPM2*, *ENO1* and *CDC19* and (**B**) *HGT2* and *HXT5* were measured by qRT-PCR and normalized to the control transcript *PMA1*. Error bars represent SEM (A: n = 3; B: n = 2); * indicates genes upregulated with *p*≤0.05 while **denotes *p*≤0.01. Unless otherwise indicated, comparisons were made to the respective wild type.

To gain additional insight into the observed changes in glycolytic intermediates, we used RT-PCR to determine if transcripts involved in glycolysis were upregulated in the *ssn3* mutant. Our results indicated majority of the glycolysis-related transcripts were expressed at higher relative levels in the absence of *SSN3* when normalized to the housekeeping transcript *PMA1* (Figure 6A). Specifically, there was a significant increase in the levels of transcripts encoding the enzymes *HXK2* (hexokinase II), *PFK1* (6-phosphofructokinase I), *FBA1* (fructose-bisphosphate adolase), *TPI1* (triose-phosphate isomerase), *PGK1* (phosphoglycerate kinase), *GPM1* (phosphoglycerate mutase I), *GPM2* (phosphoglycerate mutase II), *ENO1* (enolase) and *CDC19* (phosphoglycerate kinase). *PGI1* (phosphoglucose isomerase) and *PFK2* (6-phosphofructokinase I) were not significantly higher in the mutant.

**Figure 7:**
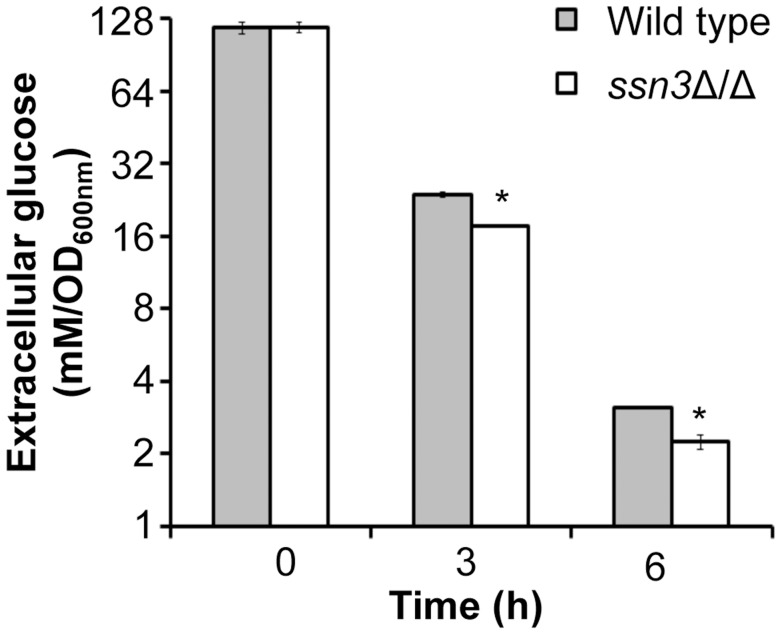
Absence of Ssn3 results in increased glucose transport. Wild type (SC5314) and *ssn3*Δ/Δ were cultured at 30°C for 6 h in YNBAG_10_P (yeast growth conditions). Supernatants were then collected for HPLC analysis of glucose consumption. All data were normalized to OD_600 nm_, and error bars represent SEM (n = 3); * indicates *p*≤0.05 compared to the wild type.

An analysis of transcripts associated with glucose transport also showed increased levels of mRNAs for two glucose transporters whose expression is associated with colony morphology [68] or biofilm growth [69]: *HXT5* and *HGT2*. Both were significantly higher in the mutant (Figure 6B) compared to both reference strains. Interestingly, van de Peppel and colleagues found that transcript levels of *HXT5* were also higher in the *S. cerevisiae* Cdk8 module mutants [38]. To directly test the hypothesis that glucose uptake and catabolism were faster in the *ssn3*Δ/Δ strain, we compared glucose consumption by the wild type and *ssn3*Δ/Δ over 12 h. Yeast growth conditions (30°C and the absence of GlcNAc) were used rather than hypha-inducing conditions in order to prevent cell clumping which could impact nutrient uptake or oxygen availability. Our analysis of extracellular glucose concentrations by high-performance liquid chromatography (HPLC) indicated that significantly higher levels of glucose remained in supernatants from wild type cultures, when compared to those from mutant cultures, at both 3 h and 6 h (Figure 7), supporting our hypothesis that the mutant consumed glucose at a faster rate than the wild type. No extracellular glucose was detected in the supernatants of the wild type or mutant cultures at later time points.

### The *ssn3* mutant has increased oxidative metabolism, but not fermentation

To further understand glucose utilization by the *ssn3* mutant, we assessed products in other pathways related to glucose catabolism. First, we determined levels of ethanol and glycerol, two *C. albicans* fermentation products. Our data revealed no notable differences in ethanol or glycerol production between the mutant and wild type strain by HPLC (Figure S6A). Intracellular glycerol levels did not differ between these strains in the metabolomics study (Table S2), and ethanol was not detected as an intracellular metabolite. Second, we assessed acidification of the extracellular milieu, indicative of acetic acid production, by the wild type and *ssn3* mutant using the pH indicator bromocresol green in medium with glucose as the sole carbon source. We found no differences in acidification levels between strains (Figure S6B). Taken together, these results suggested that the absence of Ssn3 did not promote the conversion of glucose to known fermentation products. Other glucose-related metabolic pathways were also considered. The canonical intermediates of the pentose phosphate pathway (PPP) were either decreased or unaffected by the absence of Ssn3 (Table 1). Additionally, some metabolites that are associated with the PPP, such as gluconate, were at lower levels in the *ssn3* mutant compared to the wild type or the complemented strain (Table 1). These findings suggest that glycolysis, but not all glucose-related pathways, was altered upon loss of Ssn3 function.

**Figure 8:**
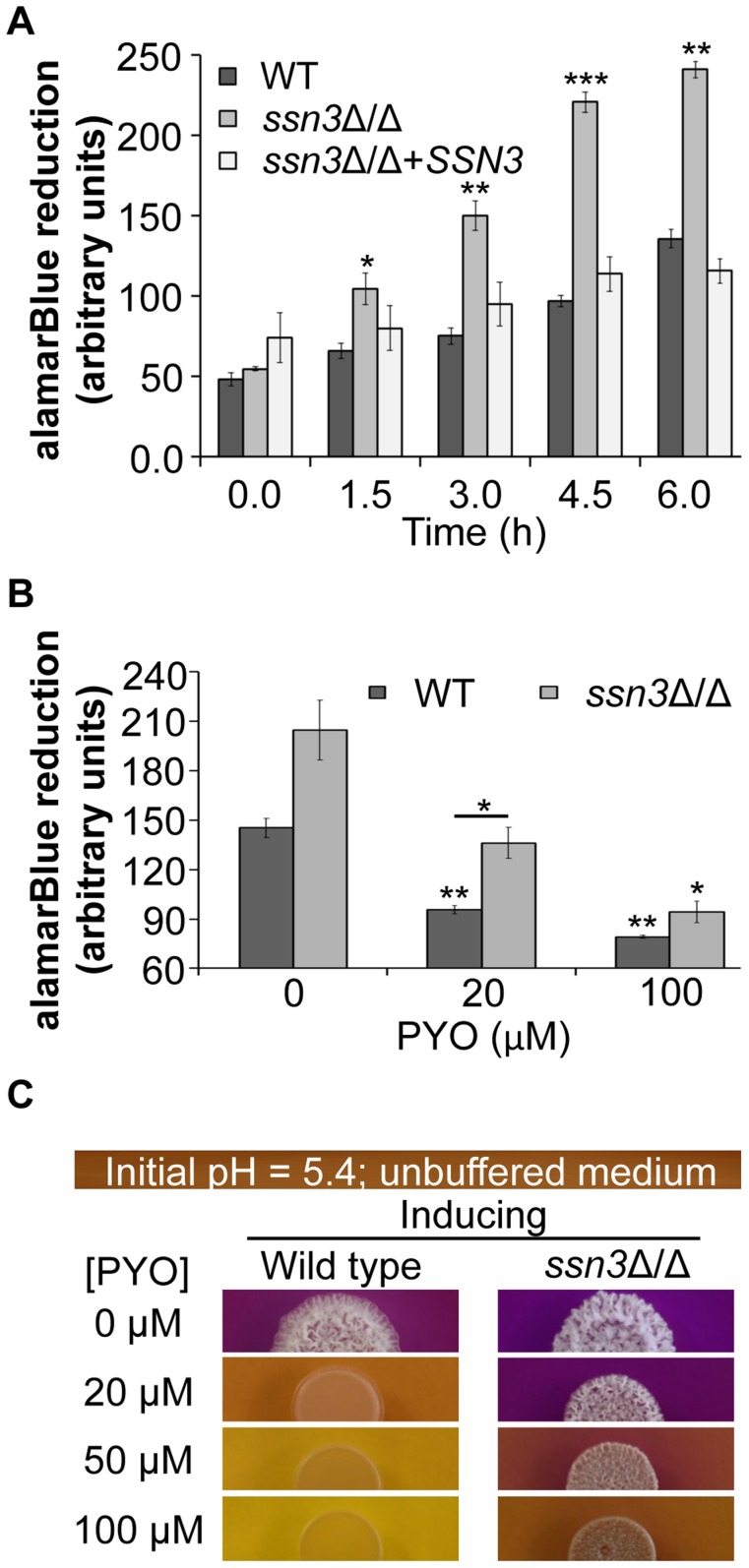
The increased oxidative metabolism of the *ssn3* mutant directly correlates with wrinkled colony formation. Wild type (WT; SC5314), *ssn3*Δ/Δ and *ssn3*Δ/Δ+*SSN3* were cultured at 30°C for 6 h in YNBG_100_P. alamarBlue reduction was measured (**A**) in the absence of treatment and (**B**) with exposure to 0, 20 or 100 µM PYO. The reduction of alamarBlue, presented as arbitrary units (AU) was normalized to the OD_600 nm_ culture as described in the Methods section. Error bars represent SEM (n = 3); * = *p*≤0.05, ** = *p*≤0.01, *** = *p*≤0.001 with respect to wild type at the respective time point (A), the vehicle-treated wild type (B) or, where indicated, the 20 µM PYO-treated wild type (B). (**C**) Colonies of wild type (SC5314) and *ssn3*Δ/Δ were grown on YNBAG_10_N-agar containing 0.01% bromocresol purple in the presence of 0, 20, 50 or 100 µM PYO for 48 h at 37°C and then imaged with a digital camera. Error bars represent SEM (n = 3); * = *p*≤0.05, ** = *p*≤0.01, *** = *p*≤0.001 with respect to wild type at the respective time point (A), the vehicle-treated wild type (B) or, where indicated, the 20 µM PYO-treated wild type (B).

Given our evidence that *ssn3*Δ/Δ acquired glucose more rapidly than the wild type, without having higher levels of fermentation products or PPP intermediates, we determined if the absence of Ssn3 increased oxidative metabolism using the reduction of alamarBlue assay as a read out [70], [71]. The same growth conditions as those in the HPLC studies were used except that glucose, at a higher concentration (100 mM), was provided as the sole carbon source. We found that the *ssn3* mutant reduced alamarBlue at a significantly faster rate than strains with functional *SSN3*, indicating that the mutant had higher levels of respiratory activity (Figure 8A). A similar phenotype was observed for the remaining Cdk8 module mutants (Figure S7). While there was a slight enhancement in early growth rates in early exponential phase cultures, the maximal growth rates and culture yields were similar for both mutant and reference strains (Figure S8).

**Figure 9:**
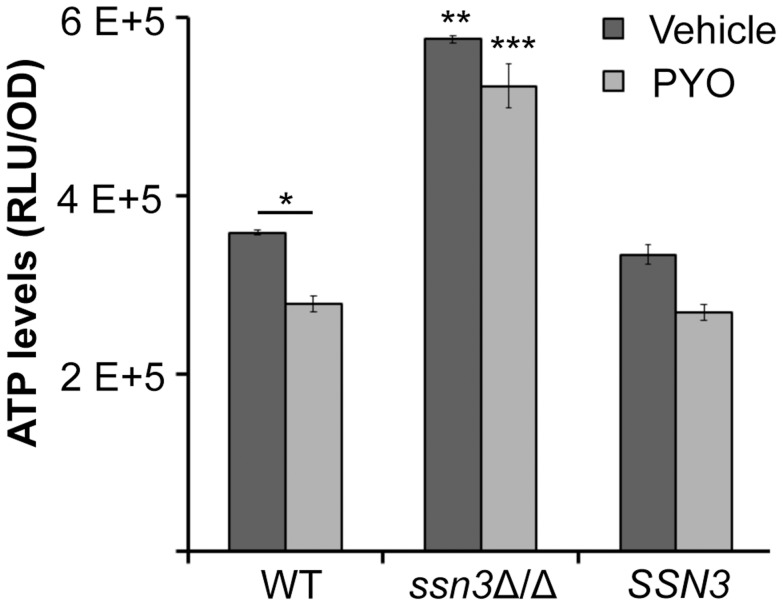
The *ssn3* mutant contains higher levels of ATP in the presence and absence of PYO. ATP levels of wild type (WT; SC5314), *ssn3*Δ/Δ (Δ/Δ) and *ssn3*Δ/Δ+*SSN3* (*SSN3*) were measured following 6 h incubation at 37°C in YNBAG_10_NP and treated with vehicle or 20 µM PYO. Error bars represent SEM (n = 3); * = *p*≤0.05, ** = *p*≤0.01 and *** = *p*≤0.001. Unless otherwise indicated, comparisons were made to the respective wild type control. ATP levels are directly proportional to the luminescent signal which is stated in relative light units (RLU) normalized to OD_600_.

Consistent with the observed elevation in oxidative metabolism upon loss of *SSN3*, there were also higher levels of ATP in the *ssn3*Δ/Δ strain in both inducing conditions comparable to those used in the genetic screen and plate-based assays (Figure 9). As in the other experiments described above, the *ssn8*, *srb8* and *srb9* mutants phenocopied the *ssn3* mutant in that they too had higher levels of ATP when compared to the wild type (Figure S9).

### Extent of metabolic inhibition by PYO correlates with morphology

PYO has been shown to interfere with components of the electron transport chain in both fungi and mammals due to its ability to accept and donate electrons [72]. Because respiratory metabolism has been linked to hyphal growth [30] and wrinkled colony formation [25], and loss of Ssn3 function results in higher basal oxidative metabolism and continued colony wrinkling in the presence of PYO, we sought to determine the effects of PYO on metabolism of the *ssn3* mutant. When we evaluated the effects of PYO on ATP levels, we found that ATP levels in the *ssn3* mutant were significantly higher than the wild type or the complemented variant in both the presence and absence of PYO (Figure 9).

To further explore the relationship between the inhibition of respiration and the inhibition of colony wrinkling, we repeated the alamarBlue assay with increasing concentrations of PYO. We found that the *ssn3* mutant grown in medium with 20 µM PYO had significantly higher levels of metabolic activity when compared to the wild type at the same concentration and, unlike the wild type, the mutant wrinkled at this concentration of PYO (Figure 8B and Figure 8C). In fact, the *ssn3* mutant treated with 20 µM PYO had respiratory activity comparable to the vehicle-treated wild type, and wrinkled colonies were formed in both cases (Figure 8B and Figure 8C). In contrast, at 100 µM PYO, there was not a significant difference in oxidative metabolism between the *ssn3* mutant and the wild type (Figure 8B), and both colonies were smooth and largely comprised of yeast with this treatment concentration (Figure 8B and Figure 8C). These data strongly support a model in which respiratory metabolism promotes, or is required, for hyphal growth, wrinkled colony formation and, likely, biofilm formation.

To determine if the increased metabolism of the mutants conferred resistance to other compounds with the potential to impact respiration, we compared the effects of other mitochondrial inhibitors on the colony morphologies and alkalinization phenotypes of the wild type and *ssn3* mutant. For these studies, we employed the use of methylene blue (MB), a sulfur-containing phenothiazine known to impair electron transfer [73], [74], and antimycin A (AA) an inhibitor of complex III of the electron transport chain. As with PYO, the *ssn3* mutant formed wrinkled colonies in the presence of 1 µM MB, while the wild type and respective complemented strain grew as smooth colonies (Figure S10A). Interestingly, while the effect of 1 µM MB and 20 µM PYO on wrinkling were comparable, PYO caused acidification of the medium while MB did not inhibit alkalinization suggesting that the metabolic response to different types of mitochondrial inhibition may differ (Figure S10A). It should be noted that the concentration of MB that inhibits wrinkling of *C. albicans* is lower than the concentration of bioavailable MB following oral administration for ailments such as malaria [75]. When the effects of AA were assessed, it was found that the growth of the mutant was much more strongly impaired than either the wild type or the complemented derivative making it challenging to assess the effects of this compound on metabolism and morphology (Figure S10B). The reason for this sensitivity is unknown, but may relate to the altered sensitivity to ROS which can be generated upon treatment with AA [76].

### The effects of PYO on the metabolic profile of *C. albicans*

To learn more about the effects of PYO on *C. albicans*, we further analyzed the metabolomics data, and made several observations. First, we obtained the reassuring result that PYO was only detected in PYO-treated cells (Table S2). Second, in accordance with previous findings that this phenazine can inhibit the activity of succinate dehydrogenase (complex II in the electron transport chain and a component of the TCA cycle) [77]), PYO significantly (p<0.05) increased the levels of succinate by 4.7-fold and 8.1-fold in the wild type and the *ssn3*Δ/Δ respectively (Table S2). Other TCA cycle intermediates (citrate, homocitrate and α-ketoglutarate) were significantly lower upon PYO treatment of both strains (Table S2) suggesting there was not a general increase in TCA cycle intermediates. Third, metabolites within the various categories were typically altered in the same direction in the wild type and the mutant (Figure S11 and Table S2). Fourth, we found that the mutant retained 40% higher glucose levels than the wild type in the presence of PYO even though PYO-treatment resulted in a 33 and 37% reduction of intracellular levels of glucose for the wild type and the *ssn3* mutant respectively. This is consistent with our model that the mutant had higher levels of glycolysis associated metabolites (Table 1) and transcripts (Figure 6), glucose consumption (Figure 7) and oxidative metabolism (Figure 8 and Figure 9) and was thus more resistant to the effects of PYO. Overall, these four results suggest the difference in PYO-sensitivity is due at least in part to differences in basal metabolism between the wild type and *ssn3* mutant, rather than an altered response to PYO.

### The *ssn3* mutant shows enhanced biofilm formation on silicone catheter material

**Figure 10:**
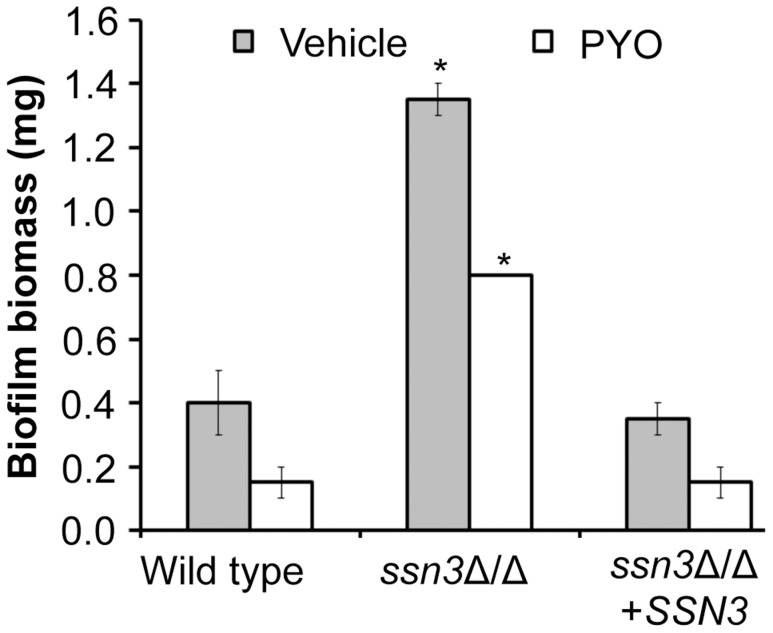
Mutation of *SSN3* increases biofilm formation. Wild type (SC5314), *ssn3*Δ/Δ and *ssn3*Δ/Δ+*SSN3* were grown at 37°C on serum-soaked silicone squares submerged in YNBG_100_. After 48 h had elapsed, biofilms were dried in an oven and then weighed. Error bars represent SEM (n = 2); * = p≤0.05 with respect to wild type.

The loss of Ssn3 and other components of the Cdk8 module of Mediator increased resistance to the repressive effect of PYO on respiratory activity and colony wrinkling. To determine if the colony phenotypes in the presence and absence of PYO extended to *ssn3*Δ/Δ biofilm phenotypes in a clinically-relevant *C. albicans* biofilm model, we assessed biofilm formation on silicone catheter material by the wild type, *ssn3*Δ/Δ and *ssn3*Δ/Δ+*SSN3* strains in medium with and without PYO. While PYO decreased biofilm formation by all strains, the PYO-treated mutant biofilms had more biomass than the PYO-treated reference strains (Figure 10). In fact, the PYO-treated *ssn3* mutant biofilms produced more biofilm than the wild type and complemented strains grown in the absence of PYO. Under the vehicle-control conditions, the *ssn3* mutant produced significantly larger biofilm biomass compared to the control strains (Figure 10).

## Discussion

The studies presented here, in conjunction with previous studies [25], [30], demonstrate positive links between metabolic activity and the induction of hyphal growth and biofilm formation programs. Here, we show that mutation of any component of the Cdk8 module of the transcriptional co-regulator Mediator results in increased metabolic activity. Using the *ssn3* mutant as a model, we also show that elevated glycolysis and respiration correlate with a need for higher concentrations of PYO to both inhibit metabolism and prevent wrinkled colony formation. Previous studies have shown that glycolysis-related genes are upregulated during biofilm formation [78]–[80]. We found these transcripts, along with the chemical intermediates in the glycolysis pathway, were also higher in the *ssn3* mutant when compared to the control strains. These differences likely contributed to the hyperwrinkly and hyperbiofilm phenotype of the mutant.

Both in *C. albicans* and other microbial species [24], wrinkled colonies have increased oxygen availability within the colony despite higher levels of respiration [25], suggesting that, like biofilms, these architectures provide a structural mechanism for gaining access to oxygen as a terminal electron acceptor and alleviating redox stress. The inhibition of wrinkled colony or biofilm development by reducing respiratory potential, without inhibiting growth due to the potential for fermentation, suggests the existence of a feedback mechanism between metabolism and cellular and colony morphology. This model is supported by work of other groups which show that biofilm formation and colony wrinkling are enhanced in low oxygen environments [7], [26], [27]. d'Enfert and colleagues [7] have shown that inactivation of *TYE7*, a positive regulator of glycolysis, results in hyperfilamentation which compromises the integrity of biofilms formed by the *tye7* mutant. It should be noted however that this phenotype was observed specifically in low oxygen environments, which is in contrast to our data which were gathered under atmospheric oxygen concentrations. This difference is likely important to consider, as the *tye7* mutant did not exhibit a hyperwrinkly phenotype in our assays or an altered response to PYO (Figure S12). We speculate that the decision to form a biofilm incorporates information on the flux through the glycolysis pathway and mitochondrial activity, and future studies will test this hypothesis.

**Figure 11:**
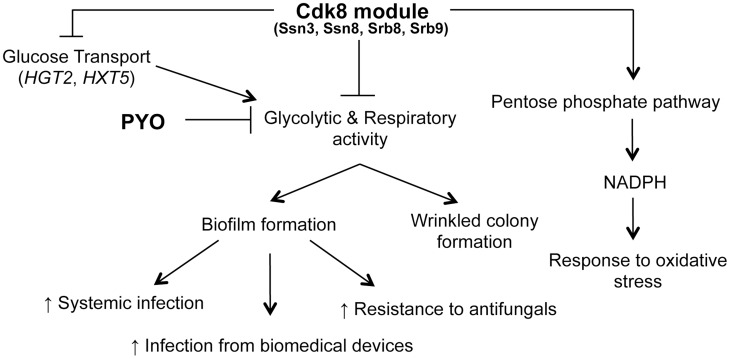
Phenotypes related to defects in the Cdk8 module can be multifactorial. The Cdk8 module of Mediator negatively regulates glucose transport, glycolysis and respiratory activity, with the latter two being exacerbated by treatment with PYO. This module positively regulates NADPH production via the PPP, which partly explains the increased sensitivity of Cdk8 mutants to oxidative stress. Ultimately, biofilm formation is associated with increased infection and adverse patient prognosis.

The direct signals between metabolism and the pathways that control filamentation and biofilm development are not yet known. However, the higher levels of ATP in the *ssn3* mutant in the presence of PYO (Figure 9) might indicate increased potential for activation of the Ras1-adenylate cyclase signaling pathway that is required for hyphal growth as ATP is the substrate for cAMP synthesis (Figure 11 [30], [81]). Consistent with a model in which cAMP signaling is elevated in the *ssn3* mutant, *HSP12* and *CTA1*, two cAMP repressed genes [82]–[84] were found to be significantly lower in the *ssn3* mutant in comparison to the wild type (Table S3). The next steps in this research will be to understand how loss of Cdk8 function influences the transcription of these and related genes. As our data indicate that metabolic activity is de-repressed upon mutation of any of the four Cdk8 module subunits or the loss of kinase activity, we propose that Ssn3 might be negatively regulating a transcriptional repressor by decreasing its stability or activity upon phosphorylation, or positively regulating a factor involved in metabolic downregulation [85]. Upon future discovery of direct targets of Cdk8 module activity, we will also be able to determine if changes in Cdk8-regulation contribute to morphological transitions and biofilm formation. In *S. cerevisiae*, the Cdk8 module has been shown to control both metabolic pathways and morphology suggesting that connections between morphology and metabolism may be common across fungi [36], [86]–[88].

We previously reported that PYO inhibits both wrinkling and alkalinization in a wild type strain of *C. albicans*, while its thioanalogue MB only inhibits wrinkling [25]. Using the Cdk8 mutants, we uncovered further evidence that all respiratory inhibitors do not exert the same effect on the behavior of *C. albicans*. Specifically, we show that the effect of MB or AA on *C. albicans* was not identical to that of PYO, as MB did not have a marked effect on alkalinization while the Cdk8 module mutants exhibited a growth defect upon treatment with AA, likely due to AA-induced generation of ROS [89]. Our observation that the Cdk8 mutants have markedly reduced growth in the presence of AA, which inhibits transfer of electrons from cytochrome b to cytochrome c of the electron transport chain without affecting other respiratory chains [89], suggests these alternate electron transport chains may be affected by absence of Ssn3. Taken together, the variation in response to PYO, MB and AA suggests *C. albicans* responds differently to different respiratory inhibitors in terms of pH, morphology and overall growth.

While catabolism of amino acids promotes hyphal growth and wrinkled colony formation by raising the extracellular pH [66], and the *ssn3* mutant was found to be a hyperalkalinizer (Figure 5 and Figure S4C), the ability of the *ssn3* mutant to filament in the presence of PYO was not attributable to medium pH differences (Figure S4A). In addition, the *ssn3* mutant was also resistant to PYO in the absence of amino acids suggesting that resistance to PYO was not due to differences in amino acid catabolism. It is, however, interesting to consider how the Cdk8 module may impact amino acid metabolism. Our metabolomics analyses found that the loss of Ssn3 impacted levels of various metabolites involved in the amino acid super pathway. Of the twenty key amino acids, only three—leucine, isoleucine and phenylalanine—were present at lower levels in the mutant compared to the control strains and 55% of the downregulated peptides contained these amino acids (Table S4). The biosynthesis of leucine and isoleucine in *S. cerevisiae* is controlled by the transcription factor Leu3 [90], [91]. Interestingly, Leu3 has been identified as a *C. albicans* pro-biofilm transcription factor, whose absence results in decreased transcription of hypha-specific genes encoding hyphal wall protein 1 (*HWP1*) and the agglutinin-like sequence protein 3 (*ALS3*) [92], [93]. Negative regulation of Leu3 by Ssn3 kinase activity could result in altered metabolite pools and increased expression of these genes, which could aid in explaining the hyperwrinkly and hyperbiofilm phenotypes of the *ssn3* mutant. Future studies will be required to explore relationships between Leu3 or other transcription factors involved in amino acid metabolism and the Cdk8 module.

By virtue of the fact that the Mediator complex is a co-regulator of transcription, its function will change based on the transcription factors that are active in a given condition. This may explain why the *srb9* mutant exhibits a filamentation and biofilm defect in nutrient-rich media [94], but not in liquid or in the defined medium used in these studies. Furthermore, while all four Cdk8 module mutants yielded identical phenotypes in our studies (Figure 2 and Figure S1) and in some *S. cerevisiae* studies, there are instances where mutation of the individual components reveal differences [38], [46], [65], [94]. The difference between the enhanced biofilm formation we observed in the *ssn3* mutant compared to the decreased biofilm formation in the *srb9* mutant observed by Uwamahoro and colleagues [94] may reflect such a difference as biofilm formation is a complex trait involving the expression of many genes. An understanding of these differences will require an analysis of the specific transcription factors with altered activity upon the alteration of Cdk8 function. All glycolysis genes that were found to be upregulated in the absence of Ssn3 (Figure 6A) are reportedly regulated by both Tye7 and Gal4 [95], and future studies will determine if these transcriptional regulators are controlled in part by some or all components of the Cdk8 module of Mediator.

Cdk8 module mutants have previously been shown to be more susceptible to hydrogen peroxide [46], [94]. Our metabolomics study revealed that the *ssn3* mutant had lower levels of PPP intermediates and their derivatives, and this pattern could reflect an altered ability to cope with oxidative stress because the PPP is an important source of NADPH for glutathione reduction during reactive oxygen species (ROS) exposure. Using the ratio of reduced glutathione (GSH) to oxidized glutathione (GSSG) as measure of redox stress [96], [97], we found no significant differences between the wild type, *ssn3* mutant, and the complemented derivative in control conditions. When the GSH∶GSSG ratios for wild type in control cultures versus those with PYO were compared, a large shift was evident (5.1±1.0 SD versus 0.56±0.07 SD), consistent with ROS being generated from PYO directly or upon altered respiration. Furthermore, the wild type had a significantly higher GSH∶GSSG ratio than the *ssn3* mutant grown with PYO (0.56±0.07 SD versus 0.32±0.08, p<0.01, n = 5) (Table S2) indicating that the mutant metabolism may confer a disadvantage during stress adaptation. This is in agreement with our finding that the mutants exhibit increased sensitivity to AA which has previously been shown to correlate with increased production of ROS and oxidation of glutathione [76].

There is a growing body of evidence showing that Ssn3 contributes to virulence in important ways. First, mutation of *SSN3* increased catheter-biofilm formation and increased resistance to the inhibitory effects of PYO on biofilm formation. These mutants may be useful in future studies to understand the link between metabolism and resistance to other agents such as antifungals. Second, the *ssn3* mutant has decreased virulence in a murine model [56]. Future studies will determine if this is due to altered *in vivo* metabolism, morphology or both. It is likely that, as in many cases, the phenotypes related to defects in the Cdk8 module are complicated, which is in accordance with the published reports that hundreds of genes can be impacted by this module of the Mediator complex in species from yeast to man [38]. Due to its ability to modulate gene regulation by altering the ability of multiple transcription factors to interact with RNA polymerase, the Cdk8 module in humans is currently being investigated as a target for chemotherapy drugs [98]–[100]. The possibility of influencing various cellular pathways in eukaryotic cells renders the Cdk8 module an attractive target for novel antifungals. Thus, future studies on the similarities and differences between human and fungal Mediator are needed.

## Materials and Methods

### Strains and growth conditions

The *C. albicans* strains used in this study are described in Table S5. All strains were streaked from −80°C onto YPD (1% yeast extract, 2% peptone, 2% glucose) plates every 8 days. Overnight cultures were grown in 5 ml of YPD, supplemented with uridine as indicated. Cultures were incubated at 30°C in a roller drum for 14 h, unless stated otherwise, and washed in distilled water (dH_2_O) prior to subsequent use. Stock solutions of pyocyanin (PYO, Cayman Chemicals), bromocresol purple (Sigma), bromocresol green (Sigma) and antimycin A (AA, Sigma) were prepared at 30 mM, 5%, 5% and 100 µM respectively in 100% ethanol. The methylene blue (MB, Fisher) stock solution of 3 mM was prepared in dH_2_O. All experiments that included PYO, AA or MB were conducted in the dark.

### Spot assays for wrinkled and smooth colony formation

For wrinkled colony formation, cells from overnight cultures resuspended in dH_2_O at an optical density at 600 nm (OD_600_) of 0.03 were spotted onto 3 ml of 2% agar containing 0.67% yeast nitrogen base medium with ammonium sulfate (YNB-agar; RPI Corp) supplemented with 10 mM glucose (YNBG_10_-agar), 5 mM N-acetylglucosamine (GlcNAc; YNBG_10_N-agar) and, where applicable, 2% [w/v] casamino acids (YNBAG_10_N-agar; BD Bacto). In the presence of amino acids (YNBAG_10_N), the medium was adjusted to an initial pH of 5.0–5.4, where any phenotype resulting from an increase in pH could be attributed to amino acid catabolism; when amino acids were excluded (YNBG_10_N), the medium was buffered to pH 7 using 1 M phosphate buffer (YNBG_10_NP), in order to assess the effects of high pH alone on morphology. The medium was amended with a final concentration of 20 µM PYO or 500 nM MB from a 30 mM or 3 mM stock solution respectively. AA was added at 75, 150 and 300 nM from the 100 µM stock while MB was added at 0.5 and 1 µM from the 3 mM stock solution. An equivalent volume of 100% ethanol (vehicle) was used as the vehicle control for PYO and AA, while water was used for assays including MB. An equivalent volume of 100% ethanol (vehicle) was used as the vehicle control for PYO and AA, while water was used for assays including MB. For those experiments designed to determine the effect of PYO or MB on extracellular pH, bromocresol purple or bromocresol green was added to the molten agar at 0.01% [v/v] of the medium. In all cases, cells were incubated at 37°C. The above medium conditions are referred to as “inducing” conditions.

“Non-inducing conditions” were used for smooth (yeast) colony growth. For these assays, cells from overnight cultures re-suspended in dH_2_O were spotted onto YNB-agar supplemented with 100 mM glucose (YNBG_100_-agar). This higher concentration of glucose, compared to the 10 mM used under inducing conditions, was utilized due to the absence of amino acids as an alternative carbon source, longer incubation times, or a combination thereof. The filamentation inducer GlcNAc was excluded from experiments conducted under non-inducing conditions. To assess the effect of PYO on extracellular pH under non-inducing conditions, cells were spot onto YNBAG_10_-agar to which bromocresol purple was added. YNBG_100_-agar and YNBAG_10_-agar were adjusted to pH 5.0–5.4, and cells were incubated at 30°C. As of this point, all incubations completed at 30°C to stimulate yeast growth are referred to as “non-inducing”.

As stated, colonies were imaged after 48 h with a Nikon SMZ1500 dissecting stereoscope at ×7.5 magnification or a digital camera. Unless otherwise noted, all spot assays were completed as at least three independent replicates and a representative data set is shown.

### Genetic screen of mutant library

A spot assay for wrinkled colony formation was used to screen approximately 1,500 mutants obtained from the Noble [42] and Mitchell [43]–[46] collections to identify PYO-resistant strains. Strains were grown under (1) inducing conditions (YNBAG_10_N-agar) in the presence of 20 µM PYO or an equivalent volume of vehicle and (2) non-inducing conditions (yeast growth; YNBG_100_-agar) in the absence of treatment for 48 h. A resistance phenotype was designated as the ability to wrinkle in the presence of PYO under inducing conditions without exhibiting wrinkling under non-inducing conditions.

### Strain construction

Histidine prototrophy was restored to the *ssn3* and *ssn8* mutants by transformation with EcoRI/NotI linearized pDDB78 plasmid (Table S5). *SSN3* and *SSN8* mutations in the auxotrophic strains were complemented with the respective native alleles, concomitant with restoration of histidine prototrophy, as described by Blankenship *et al.* [46] using the plasmids and primers shown in Tables S5 and S6.

C-terminal tagging of *Candida albicans* proteins with 3X hemagglutinin (HA) epitope was performed as previously described [101]. Briefly, to construct *SSN3-3HA/SSN3-3HA* (DH2209), the two copies of *SSN3* were sequentially 3XHA tagged by integrating DNA cassettes amplified by the primers of *SSN3* tag sense/*SSN3* tag anti from pFA-3XHA-HIS1 and pFA-3XHA-SAT1 in frame with the 3′ end of the *SSN3* ORF. To construct *SSN8-3HA/SSN8-3HA* (DH2216), the two copies of *SSN8* were 3XHA tagged by integrating DNA cassettes amplified by the primers of *SSN8* tag sense/*SSN8* tag anti from pFA-3XHA-HIS1 and pFA-3XHA-ARG4 in frame with the 3′ end of *SSN8* ORF. Both strains had wild-type sensitivity to PYO under wrinkling-inducing conditions.

### Western blot

Overnight YPD cultures (supplemented with 0.2 mM uridine) were washed, re-suspended and inoculated to an OD_600_ of 0.05 into YNBG_10_NP supplemented with uridine, arginine and histidine (0.67% YNB, 0.2% glucose, 5 mM GlcNAC 25 mM phosphate buffer pH 7.0, 0.2 mM uridine, 20 mg/L histidine, 20 mg/L arginine) treated with 20 µM PYO or an equivalent volume of vehicle. Cells were grown at 37°C (175 rpm) for 6–7 hours before collection. In this period, cultures underwent approximately 3.5 cell divisions, which was estimated by the 10-fold cell density increase (from OD 0.05 to OD 0.5) of parallel cultures grown in YNBG_10_ (YNBG_10_N without GlcNAC) at 30°C. At this time, approximately 5 OD of cells (≈10 ml) were collected, washed in cold water, pelleted in 1.5 ml screw-top tube and flash-frozen in liquid nitrogen. To lyse the cells, the pellets were re-suspended in 50 µl ESB (80 mM Tris-HCl pH 6.8, 2% sodium dodecyl sulfate (SDS), 1.5% dithiothreitol, 10% glycerol and 0.1 mg/ml bromophenol blue), boiled for 3 minutes then bead-beaten for 4 minutes by mini-beadbeater (BIOSPEC). An additional 70 µl of ESB buffer was added to each sample, which was then boiled for an additional 2 minutes. Approximately 5 µl of the resultant whole cell extract was resolved by 10% SDS polyacrylamide gel electrophoresis (SDS-PAGE), transferred to a polyvinylidene fluoride (PVDF) membrane (Millipore) and probed by rat anti-HA antibody (Roche). Goat anti-rat IgG AP conjugate (Santa Cruz) was used as the secondary antibody. The signal was developed against enhanced chemifluorescence substrate (General Electric) and scanned using Typhoon Molecular Imager (Molecular Dynamics). ECF substrates for developing HA signals were washed away by following the PVDF membrane manual (Millipore) and the same membrane was re-probed by rat anti-tubulin alpha (AbD SeroTec) and goat anti-rat IgG AP conjugate to monitor tubulin contents. Western blot signal volume was quantified by ImageQuant (Molecular Dynamics) and HA/Tubulin signal ratio of each PYO treated sample was normalized against its untreated control.

### Metabolomics

Spot assays were completed as previously described on YNBG_10_NP-agar and incubated at 37°C for 24 h. Cells were harvested, by scraping colonies from the surface of the agar using a coverslip, and then snap-frozen in an ethanol/dry ice bath. A total of 5 biological replicates were submitted to Metabolon for metabolite profiling, by GC/MS and LC/MS, of vehicle-treated wild type, *ssn3*Δ/Δ and *ssn3*Δ/Δ+*SSN3* as well as 20 µM PYO-treated wild type and *ssn3*Δ/Δ.

### High performance liquid chromatography (HPLC) analysis of glucose consumption and fermentation product formation

Cells from exponential phase cultures were washed and then inoculated to an OD_600_ of 0.05 in 5 ml YNBAG_10_P. Cultures were incubated at 30°C in a roller drum for 3 h, pelleted and then re-suspended in 5 ml of dH_2_O. The cell suspension was added at an equal volume to a microtiter dish containing 2X YNBAG_10_P. Absorbance was measured at 30°C every hour for 6 h using a SpectraMax M5 spectrophotometer. After 6 h had elapsed, 200 µl of the yeast culture was removed from the microtiter dish for filtration and HPLC analysis using the protocol previously described by Morales and colleagues [25]. Two independent experiments with 2–3 biological replicates were performed and the combined results, normalized to OD_600_, are shown.

### alamarBlue assay for mitochondrial activity

Overnight cultures re-suspended in dH_2_O were pelleted and then re-suspended in a volume of YNBG_100_P equivalent to the original volume of dH_2_O. The OD_600_ was then measured and 100 µl of fresh medium was inoculated to a final OD of 0.1 in a microtiter dish. Next, 10 µl of alamarBlue (AB; Invitrogen) was added to each well. Where applicable, PYO was added at a final concentration of 20 or 100 µM and vehicle was added at the same volume as the latter concentration. Duplicate wells were prepared to which dH_2_O was added instead of AB. The absorbance of the cultures at 30°C was measured at 570 and 600 nm, as recommended by the manufacturer, every 1.5 h over a 6 h period using a SpectraMax M5 spectrophotometer (Molecular Devices). The reduction of AB was calculated as described by the manufacturer and all data were normalized to the OD_600_ of cultures grown in the absence of AB. The data shown are representative of at least three independent replicates that each included three technical replicates.

### ATP quantification

Overnight cultures re-suspended in dH_2_O were pelleted and then re-suspended to an OD_600_ of 0.3 in 5 ml of YNBAG_10_NP and treated with either 20 µM PYO or an equivalent volume of vehicle. Cultures were incubated at 37°C (inducing). After 6 h had elapsed, ATP levels were measured using a CellTiter-Glo Luminescent Cell Viability Assay (Promega). The luminescent signal, which is proportional to ATP levels, was measured using a Tecan Infinite 200 Pro equipped with Magellan software (Tecan). All data were normalized to the OD_600_ at 6 h. Three independent replicates, each including three technical replicates, were conducted and a representative data set is presented. For ATP measurements under non-inducing conditions, strains were cultured in YNBG_100_P.

### RT-PCR

Colonies grown under wrinkling inducing conditions for 24 h on unbuffered YNBAG_10_N-agar were harvested using the protocol previously described for the metabolomics study. Immediately afterwards, RNA isolation, DNase treatment, cDNA synthesis and RT-PCR were carried out as previously described by Davis-Hanna and colleagues [82], except cDNA was synthesized using 1 µg of RNA. Transcript levels were measured for *HGT2* and *HXT5*, which encode glucose transporters [102], [103]; *HXK2*, *PGI1*, *PFK1*, *PFK2*, *FBA1*, *TPI1*, *PGK1*, *GPM1*, *GPM2*, *ENO1* and *CDC19* which encode glycolytic enzymes; and *HSP12* and *CTA1* which encode oxidative stress response genes, using a 7500 Fast Real-time PCR System (Bio-Rad) with the following thermocycler conditions: 95°C for 3 min, 95°C for 10 s, 58°C for 30 s for 34 cycles. A total of two biological replicates were completed and each included three technical replicates. All transcript levels were normalized to *PMA1* which encodes a plasma membrane protein. All primers are shown in Table S6.

### Catheter-biofilm assay

Biofilms were grown on silicone squares (Bentec Medical, Inc.) using the protocol described by Desai and colleagues [104], except YNBG_10_P and treated with 20 µM PYO or an equivalent volume of vehicle was used as the growth medium. Biomass of biofilms was determined as previously described [105].

## Supporting Information

Figure S1 Cdk8 module *srb8* and *srb9* mutants, but not Core Module mutants, exhibit increased PYO resistance. Colonies of (**A**) *srb8*Δ/Δ, *srb8*Δ/Δ+*SRB8*, *srb9*Δ/Δ and *srb9*Δ/Δ+*SRB9* and (**B**) the indicated core module mutants were grown on YNBAG_10_N-agar containing vehicle or 20 µM PYO at 37°C for 48 h and then imaged with a dissecting stereoscope. Data are representative of at least 2 independent replicates.
(TIF)

Figure S2 The *nrg1* and *tup1* mutants are not resistant to PYO. Colonies of wild type (SC5314), *nrg1*Δ/Δ and *tup1*Δ/Δ were grown on YNBAG_10_N-agar for 48 h at 37°C in the presence of vehicle or 20 µM PYO and then imaged with a dissecting stereoscope.
(TIF)

Figure S3 Mutation within the Cdk8 module increases onset of alkalinization and wrinkling. (**A**) Colonies of wild type (SC5314), *ssn3*Δ/Δ, and *ssn3*Δ/Δ+*SSN3* were grown on YNBAG_10_N-agar containing 0.01% bromocresol purple for 6 h at 37°C and then imaged with a digital camera. (**B**) Colonies of wild type (SC5314), *ssn3*Δ/Δ, *ssn8*Δ/Δ, *srb8*Δ/Δ, and *srb9*Δ/Δ were grown on YNBAG_10_N-agar containing 0.01% bromocresol purple in the presence of vehicle or 20 µM PYO for 48 h at 37°C and then imaged with a digital camera.
(TIF)

Figure S4 PYO effects on metabolism are independent of morphology and pH. Colonies of wild type (SC5314), *ssn3*Δ/Δ and *ssn3*Δ/Δ+*SSN3* were grown on YNBG_10_-agar containing 0.01% bromocresol purple in the presence of vehicle or 20 µM PYO for 48 h before imaging. Colonies were grown at 37°C on medium amended with GlcNAc and buffered to pH 7 (**A**) in the presence and (**B**) in the absence of amino acids. (**C**) Colonies were grown at 30°C on unbuffered medium amended with amino acids.
(TIF)

Figure S5 Differences in metabolites within major categories upon mutation of *SSN3*. Percent of metabolites within the indicated categories that showed significantly different levels when the *ssn3*Δ/Δ was compared to the wild type (SC5314). The numbers in parentheses represent the total number of metabolites within each category that showed a statistically significant difference (higher in red, lower in green) for the mutant when compared to the wild type or the *ssn3*Δ/Δ+*SSN3*. The metabolite identities within each category are detailed in Table S2. ^1^Cof. & Vit. = Cofactors and vitamins.
(TIF)

Figure S6 Absence of Ssn3 does not increase fermentation. (**A**) HPLC analysis of ethanol (grey bars) and glycerol (white bars) in supernatants from the wild type (SC5314) and *ssn3*Δ/Δ, cultured at 30°C for 6 h in YNBAG_10_P. All data were normalized to cell density. Error bars represent SEM (n = 3). (**B**) Colonies of wild type (SC5314) and *ssn3*Δ/Δ were grown on YNBAG_10_P-agar containing 0.01% bromocresol green for 6 h at 30°C and then imaged with a digital camera.
(TIF)

Figure S7 The *ssn8*, *srb8* and *srb9* mutants have increased oxidative metabolism. Wild type (SC5314), *ssn8*Δ/Δ, *srb8*Δ/Δ and *srb9*Δ/Δ were cultured at 30°C for 6 h in YNBG_100_P. alamarBlue reduction was measured in the absence of treatment and was normalized to the respective OD_600 nm_ culture.
(TIF)

Figure S8 Loss of Ssn3 does not increase growth rate. Wild type (SC5314), *ssn3*Δ/Δ and *ssn3*Δ/Δ+*SSN3* were grown in (**A**) YNBG_100_ or (**B**) YNBAG_10_ at 30°C with the OD_600_ measured every hour for 15 h.
(TIF)

Figure S9 The *ssn8*, *srb8* and *srb9* mutants contain higher levels of ATP than the wild type. ATP levels of wild type (WT; SC5314), *ssn8*Δ/Δ, *srb8*Δ/Δ and *srb9*Δ/Δ were measured over 4 h of incubation at 37°C in YNBAG_10_NP. ATP levels are directly proportional to the luminescent signal which is stated in relative light units (RLU).
(TIF)

Figure S10 Loss of *SSN3* increases resistance to MB, but increases sensitivity to AA. Colonies of wild type (SC5314), *ssn3*Δ/Δ, and *ssn3*Δ/Δ+*SSN3* were grown on YNBAG_10_N-agar containing 0.01% bromocresol purple in the presence of vehicle or the indicated concentrations of MB (A) or AA (B) for 48 h before imaging.
(TIF)

Figure S11 Differences in metabolites within major categories upon mutation of *SSN3* and treatment with PYO. Heat map showing the percent of metabolites that increased or decreased upon exposure to PYO within the major categories relevant to this work in the wild-type WT (SC5314) and *ssn3*Δ/Δ strains. The metabolite identities within each category are detailed in Table S2.
(TIFF)

Figure S12 Altered expression of *TYE7* does not influence wrinkling, alkalinization or sensitivity to PYO. Wrinkling, alkalinization and sensitivity to PYO were compared for *tye7*Δ/Δ, *tye7*Δ/Δ+*TYE7* and *tye7*Δ/Δ+*TYE7-OE*. Colonies were grown on YNBAG_10_N with vehicle or 20 µM PYO at 37°C for 48 h and then imaged using a digital camera.
(TIF)

Table S1 Mutants with increased resistance to the inhibitory effect of PYO on wrinkling.
(DOCX)

Table S2 Complete metabolite profile dataset.
(XLSX)

Table S3 Absence of Ssn3 results in decreased expression of *HSP12* and *CTA1*.
(DOCX)

Table S4 Absence of Ssn3 results in decreased levels of three of the twenty key amino acids.
(DOCX)

Table S5 Strains and plasmids used in this study.
(DOCX)

Table S6 Primers used in this study.
(DOCX)
