# Supplementary figures and images for: Analysis of Candida albicans Mutants Defective in the Cdk8 Module of Mediator Reveal Links between Metabolism and Biofilm Formation
Source: PLoS Genet. 2014 Oct 2;10(10):e1004567. doi: 10.1371/journal.pgen.1004567 (PMC4183431; doi:10.1371/journal.pgen.1004567)

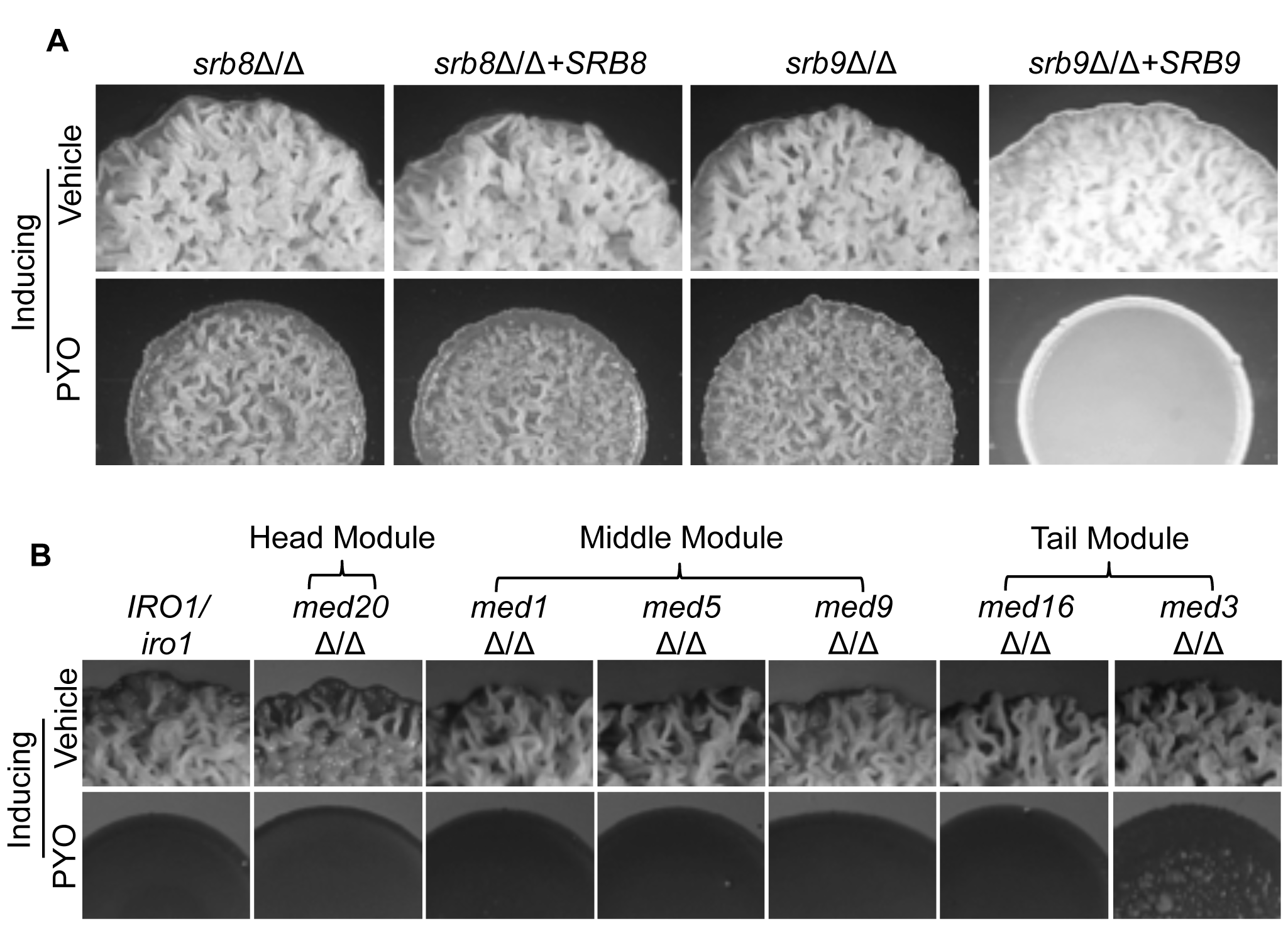

Supplement: Figure S1 — Cdk8 module srb8 and srb9 mutants, but not Core Module mutants, exhibit increased PYO resistance. Colonies of (A) srb8Δ/Δ, srb8Δ/Δ+SRB8, srb9Δ/Δ and srb9Δ/Δ+SRB9 and (B) the indicated core module mutants were grown on YNBAG10N-agar containing vehicle or 20 µM PYO at 37°C for 48 h and then imaged with a dissecting stereoscope. Data are representative of at least 2 independent replicates. (TIF) [file pgen.1004567.s001.tif]

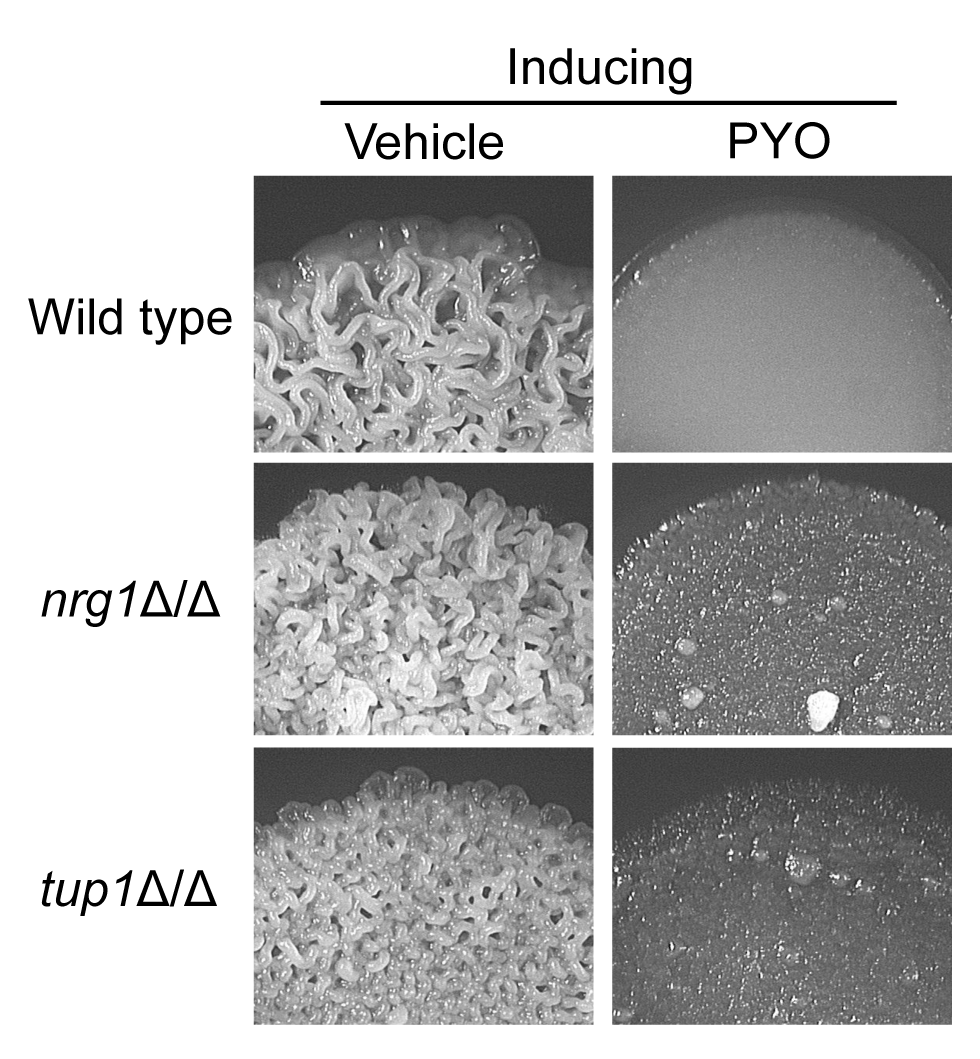

Supplement: Figure S2 — The nrg1 and tup1 mutants are not resistant to PYO. Colonies of wild type (SC5314), nrg1Δ/Δ and tup1Δ/Δ were grown on YNBAG10N-agar for 48 h at 37°C in the presence of vehicle or 20 µM PYO and then imaged with a dissecting stereoscope. (TIF) [file pgen.1004567.s002.tif]

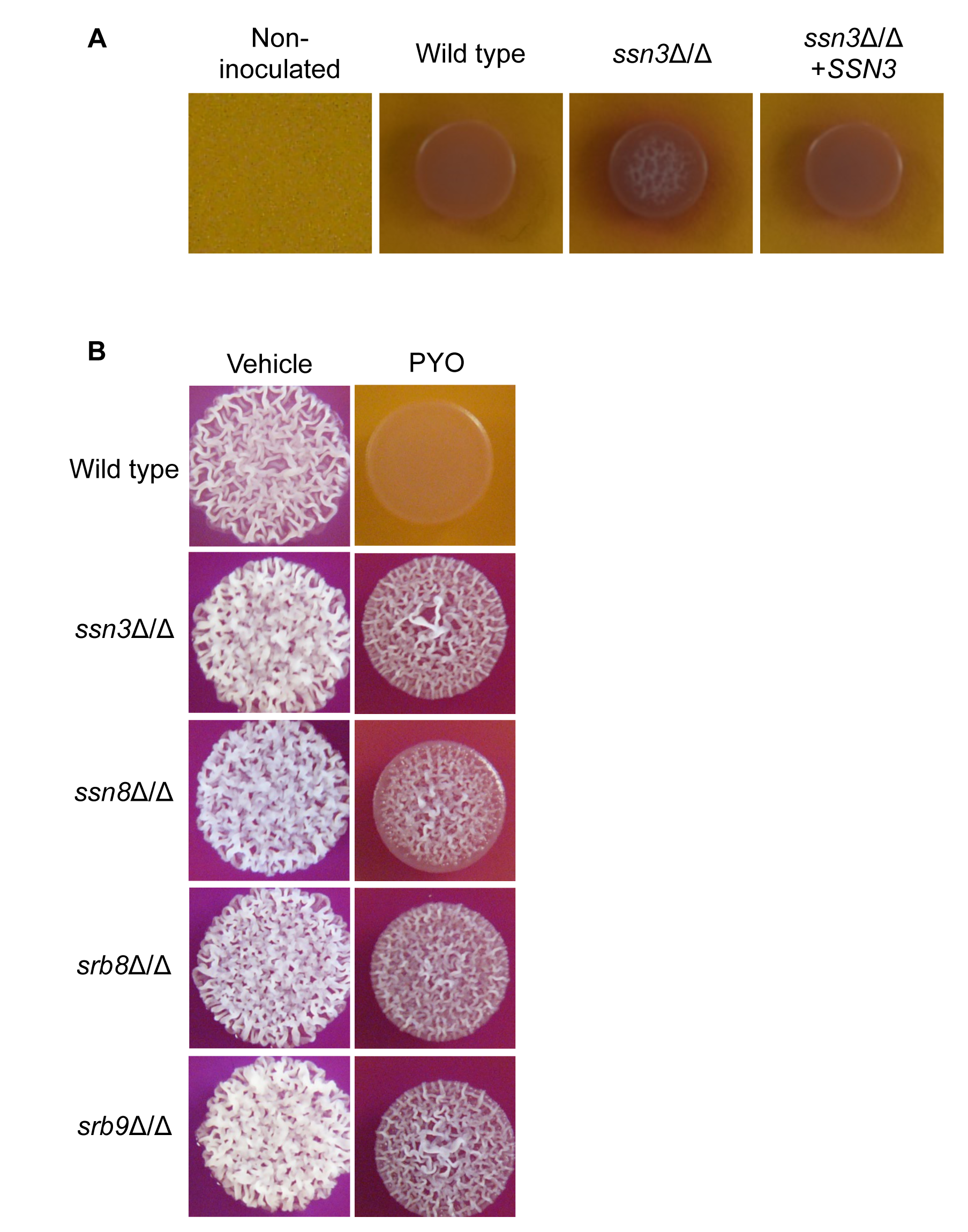

Supplement: Figure S3 — Mutation within the Cdk8 module increases onset of alkalinization and wrinkling. (A) Colonies of wild type (SC5314), ssn3Δ/Δ, and ssn3Δ/Δ+SSN3 were grown on YNBAG10N-agar containing 0.01% bromocresol purple for 6 h at 37°C and then imaged with a digital camera. (B) Colonies of wild type (SC5314), ssn3Δ/Δ, ssn8Δ/Δ, srb8Δ/Δ, and srb9Δ/Δ were grown on YNBAG10N-agar containing 0.01% bromocresol purple in the presence of vehicle or 20 µM PYO for 48 h at 37°C and then imaged with a digital camera. (TIF) [file pgen.1004567.s003.tif]

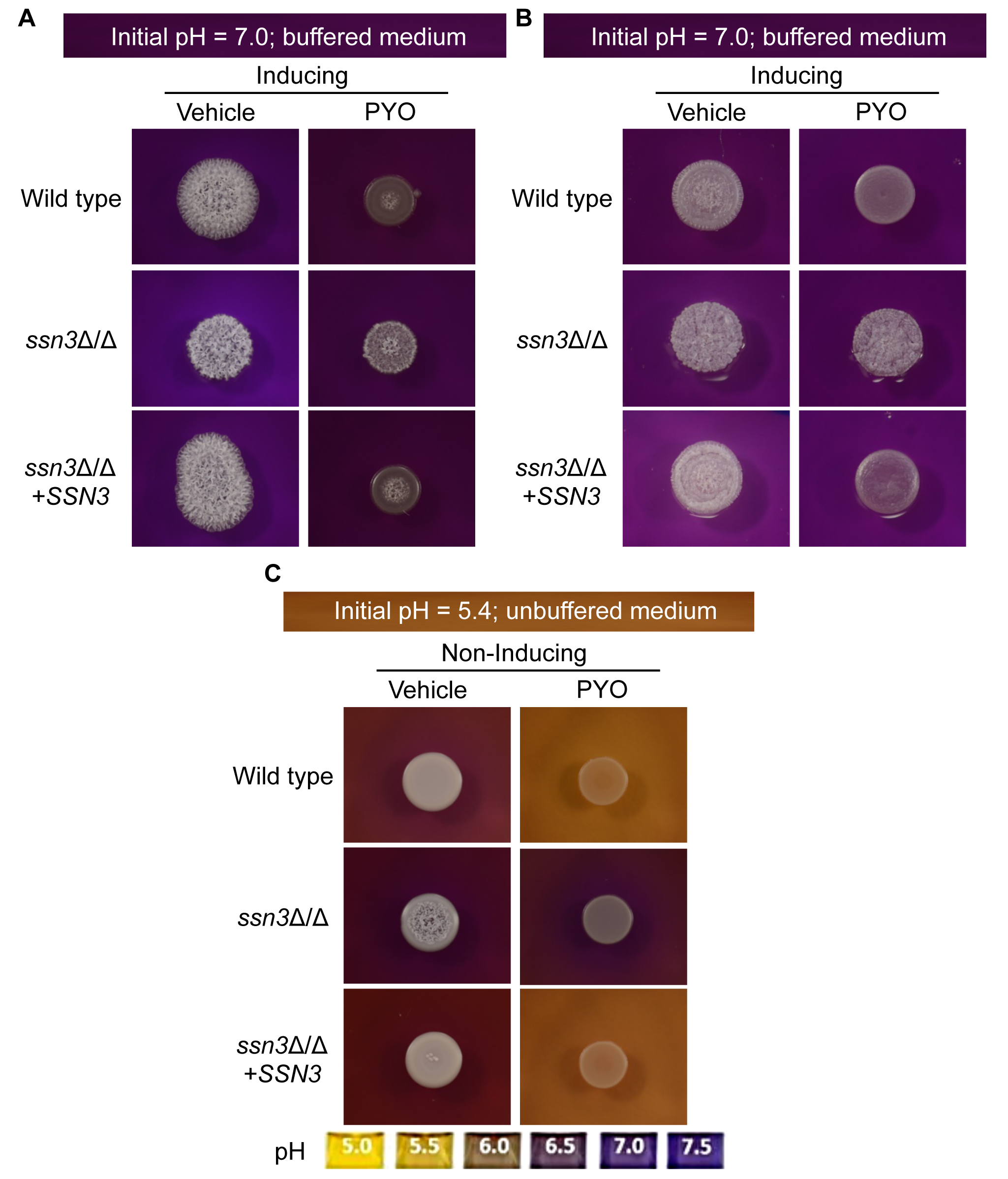

Supplement: Figure S4 — PYO effects on metabolism are independent of morphology and pH. Colonies of wild type (SC5314), ssn3Δ/Δ and ssn3Δ/Δ+SSN3 were grown on YNBG10-agar containing 0.01% bromocresol purple in the presence of vehicle or 20 µM PYO for 48 h before imaging. Colonies were grown at 37°C on medium amended with GlcNAc and buffered to pH 7 (A) in the presence and (B) in the absence of amino acids. (C) Colonies were grown at 30°C on unbuffered medium amended with amino acids. (TIF) [file pgen.1004567.s004.tif]

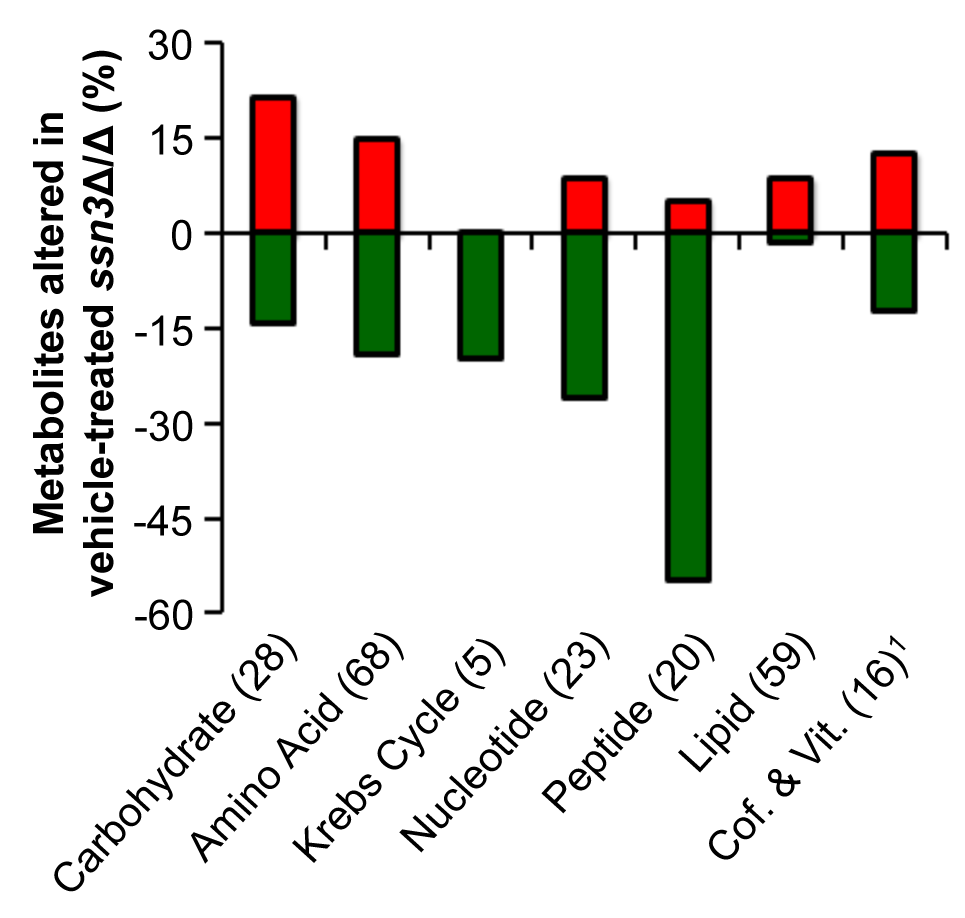

Supplement: Figure S5 — Differences in metabolites within major categories upon mutation of SSN3. Percent of metabolites within the indicated categories that showed significantly different levels when the ssn3Δ/Δ was compared to the wild type (SC5314). The numbers in parentheses represent the total number of metabolites within each category that showed a statistically significant difference (higher in red, lower in green) for the mutant when compared to the wild type or the ssn3Δ/Δ+SSN3. The metabolite identities within each category are detailed in Table S2. 1Cof. & Vit. = Cofactors and vitamins. (TIF) [file pgen.1004567.s005.tif]

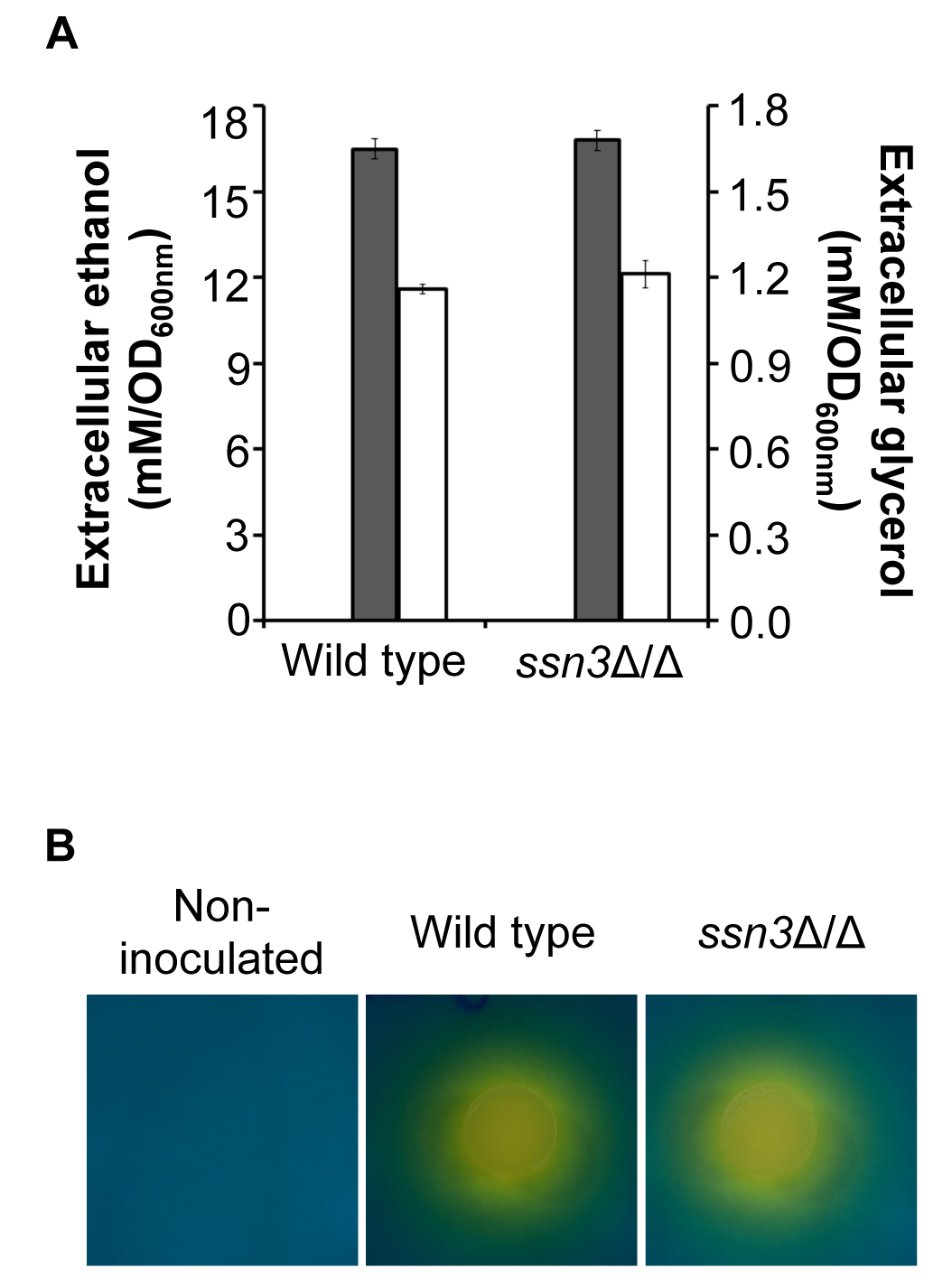

Supplement: Figure S6 — Absence of Ssn3 does not increase fermentation. (A) HPLC analysis of ethanol (grey bars) and glycerol (white bars) in supernatants from the wild type (SC5314) and ssn3Δ/Δ, cultured at 30°C for 6 h in YNBAG10P. All data were normalized to cell density. Error bars represent SEM (n = 3). (B) Colonies of wild type (SC5314) and ssn3Δ/Δ were grown on YNBAG10P-agar containing 0.01% bromocresol green for 6 h at 30°C and then imaged with a digital camera. (TIF) [file pgen.1004567.s006.tif]

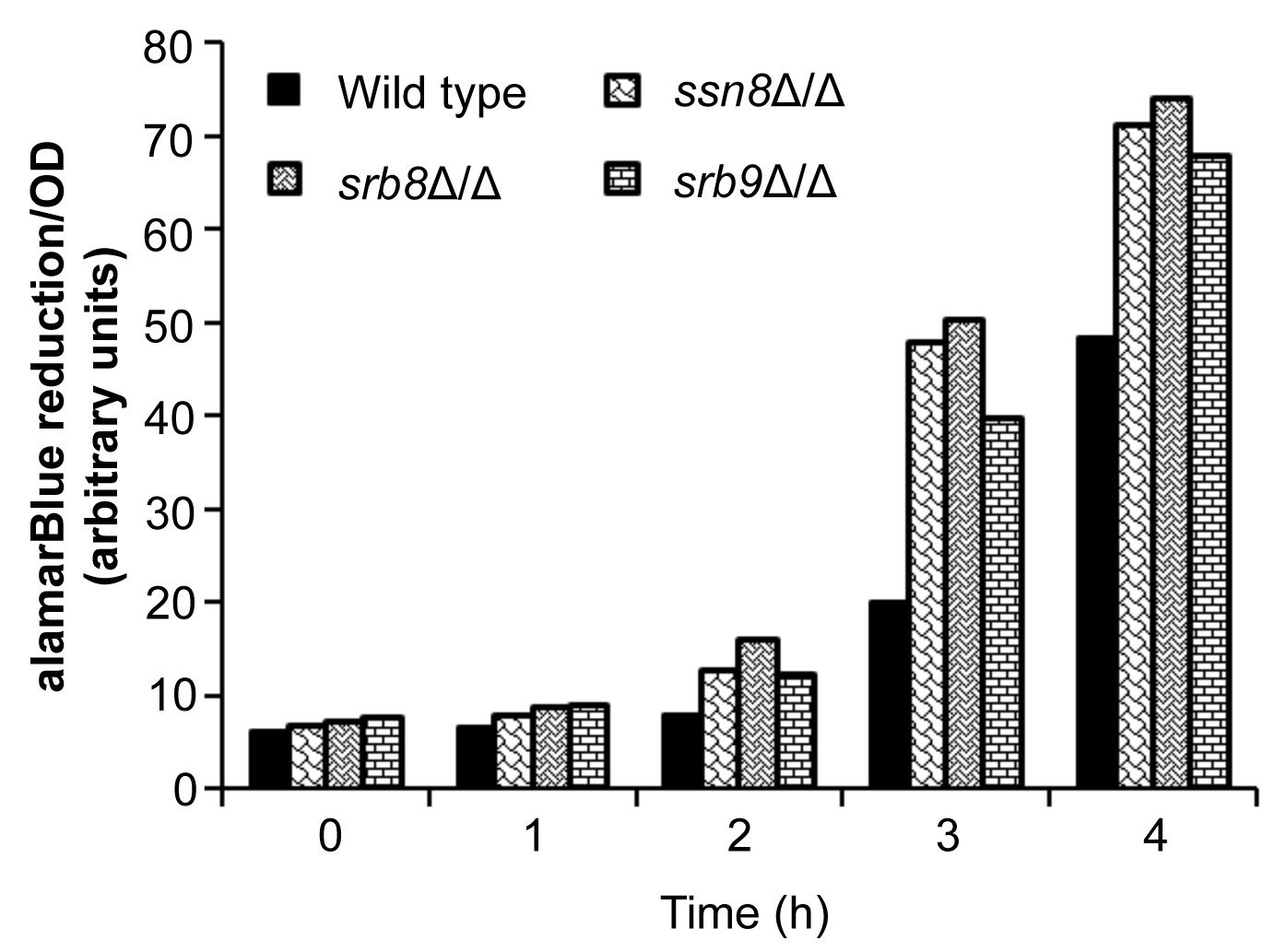

Supplement: Figure S7 — The ssn8, srb8 and srb9 mutants have increased oxidative metabolism. Wild type (SC5314), ssn8Δ/Δ, srb8Δ/Δ and srb9Δ/Δ were cultured at 30°C for 6 h in YNBG100P. alamarBlue reduction was measured in the absence of treatment and was normalized to the respective OD600 nm culture. (TIF) [file pgen.1004567.s007.tif]

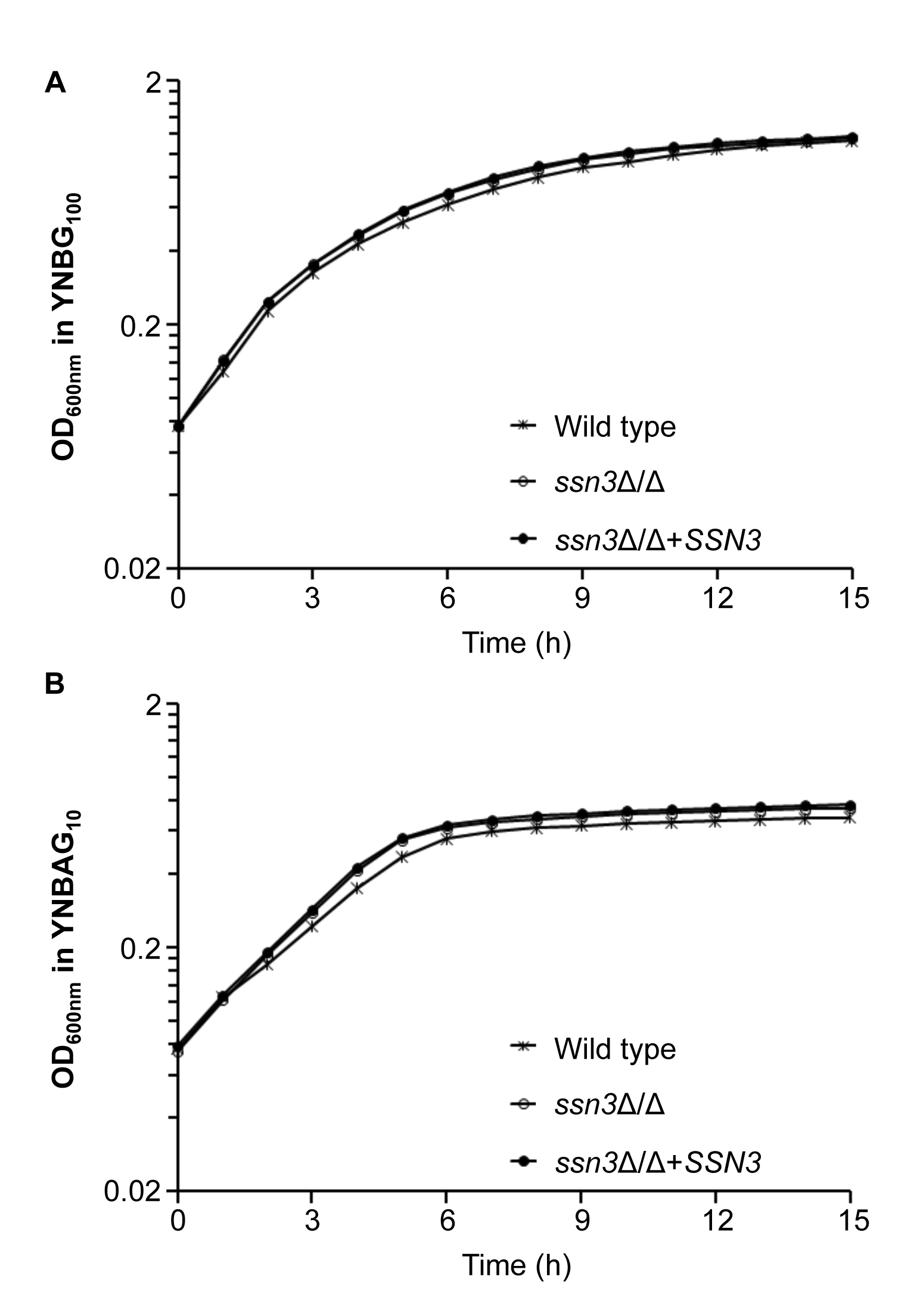

Supplement: Figure S8 — Loss of Ssn3 does not increase growth rate. Wild type (SC5314), ssn3Δ/Δ and ssn3Δ/Δ+SSN3 were grown in (A) YNBG100 or (B) YNBAG10 at 30°C with the OD600 measured every hour for 15 h. (TIF) [file pgen.1004567.s008.tif]

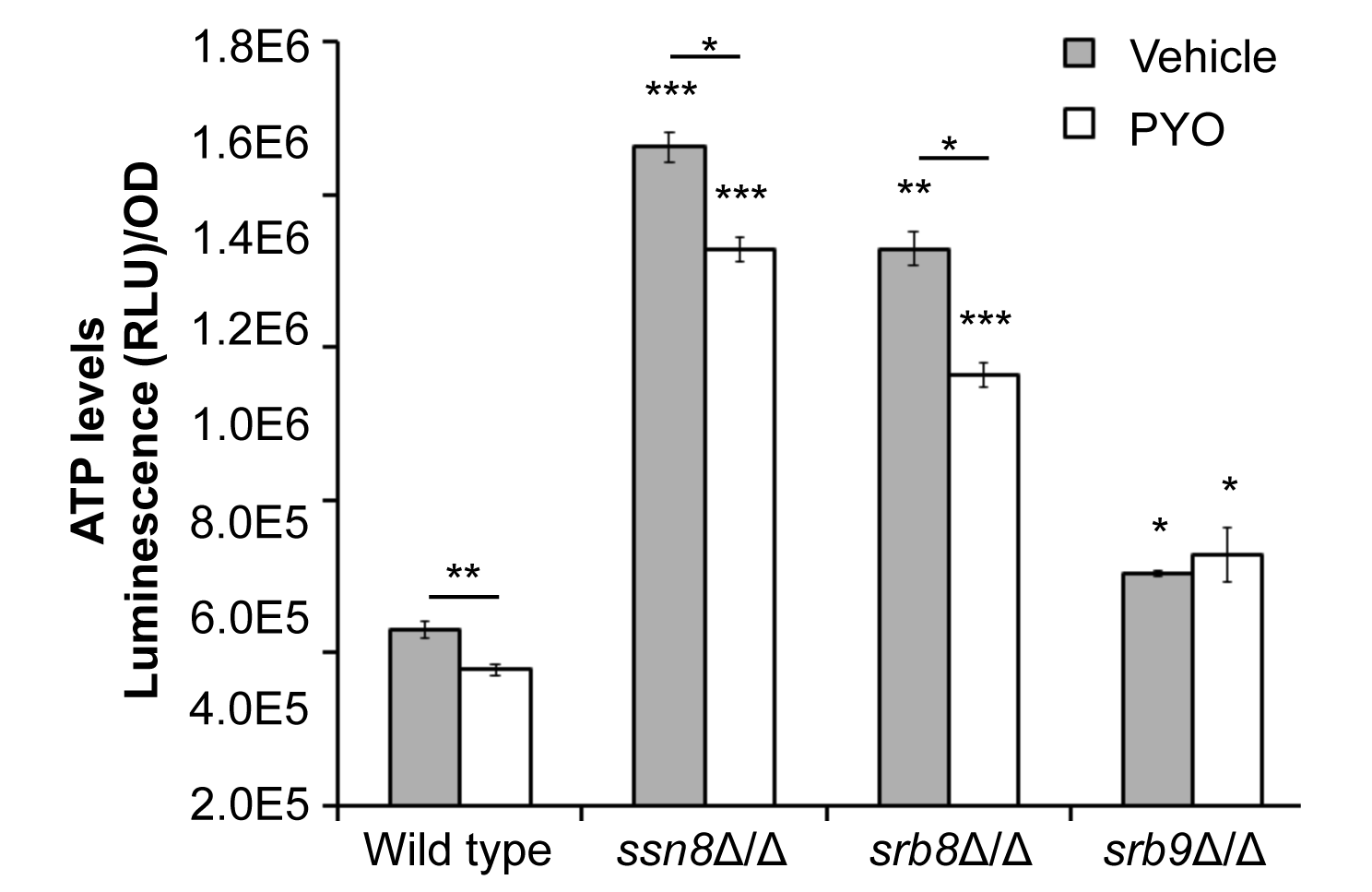

Supplement: Figure S9 — The ssn8, srb8 and srb9 mutants contain higher levels of ATP than the wild type. ATP levels of wild type (WT; SC5314), ssn8Δ/Δ, srb8Δ/Δ and srb9Δ/Δ were measured over 4 h of incubation at 37°C in YNBAG10NP. ATP levels are directly proportional to the luminescent signal which is stated in relative light units (RLU). (TIF) [file pgen.1004567.s009.tif]

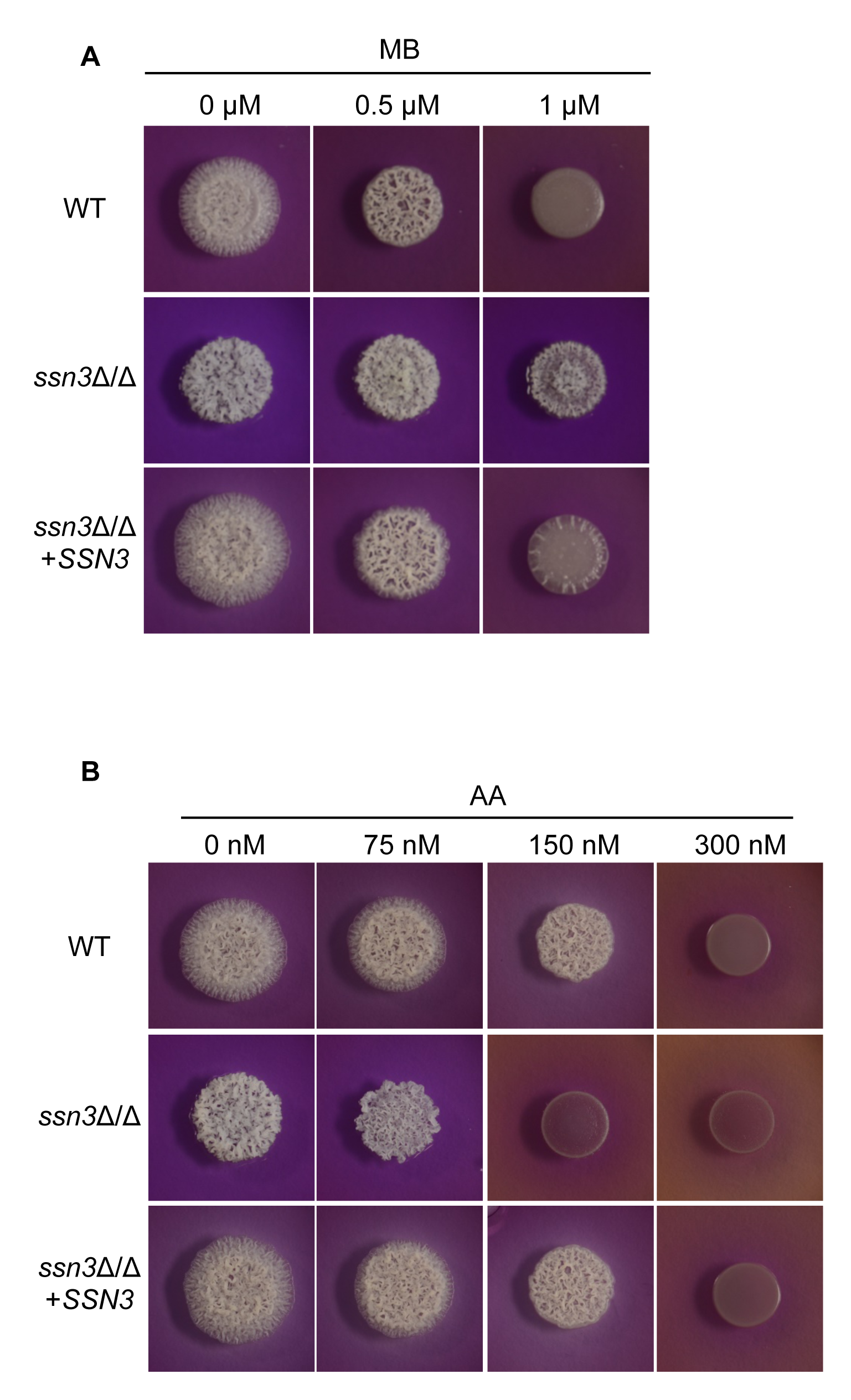

Supplement: Figure S10 — Loss of SSN3 increases resistance to MB, but increases sensitivity to AA. Colonies of wild type (SC5314), ssn3Δ/Δ, and ssn3Δ/Δ+SSN3 were grown on YNBAG10N-agar containing 0.01% bromocresol purple in the presence of vehicle or the indicated concentrations of MB (A) or AA (B) for 48 h before imaging. (TIF) [file pgen.1004567.s010.tif]

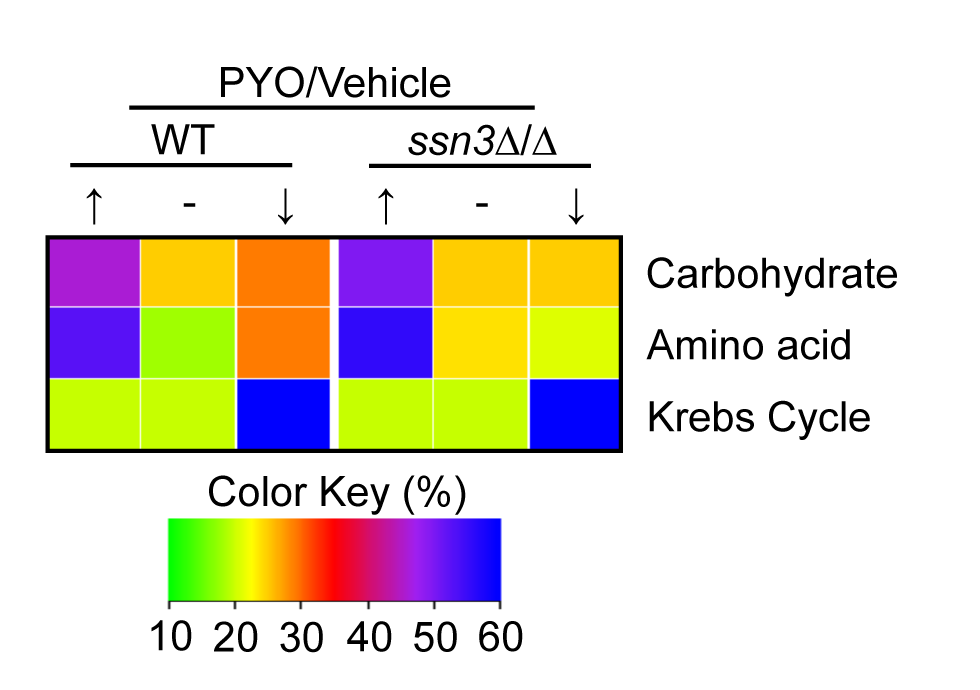

Supplement: Figure S11 — Differences in metabolites within major categories upon mutation of SSN3 and treatment with PYO. Heat map showing the percent of metabolites that increased or decreased upon exposure to PYO within the major categories relevant to this work in the wild-type WT (SC5314) and ssn3Δ/Δ strains. The metabolite identities within each category are detailed in Table S2. (TIFF) [file pgen.1004567.s011.tiff]

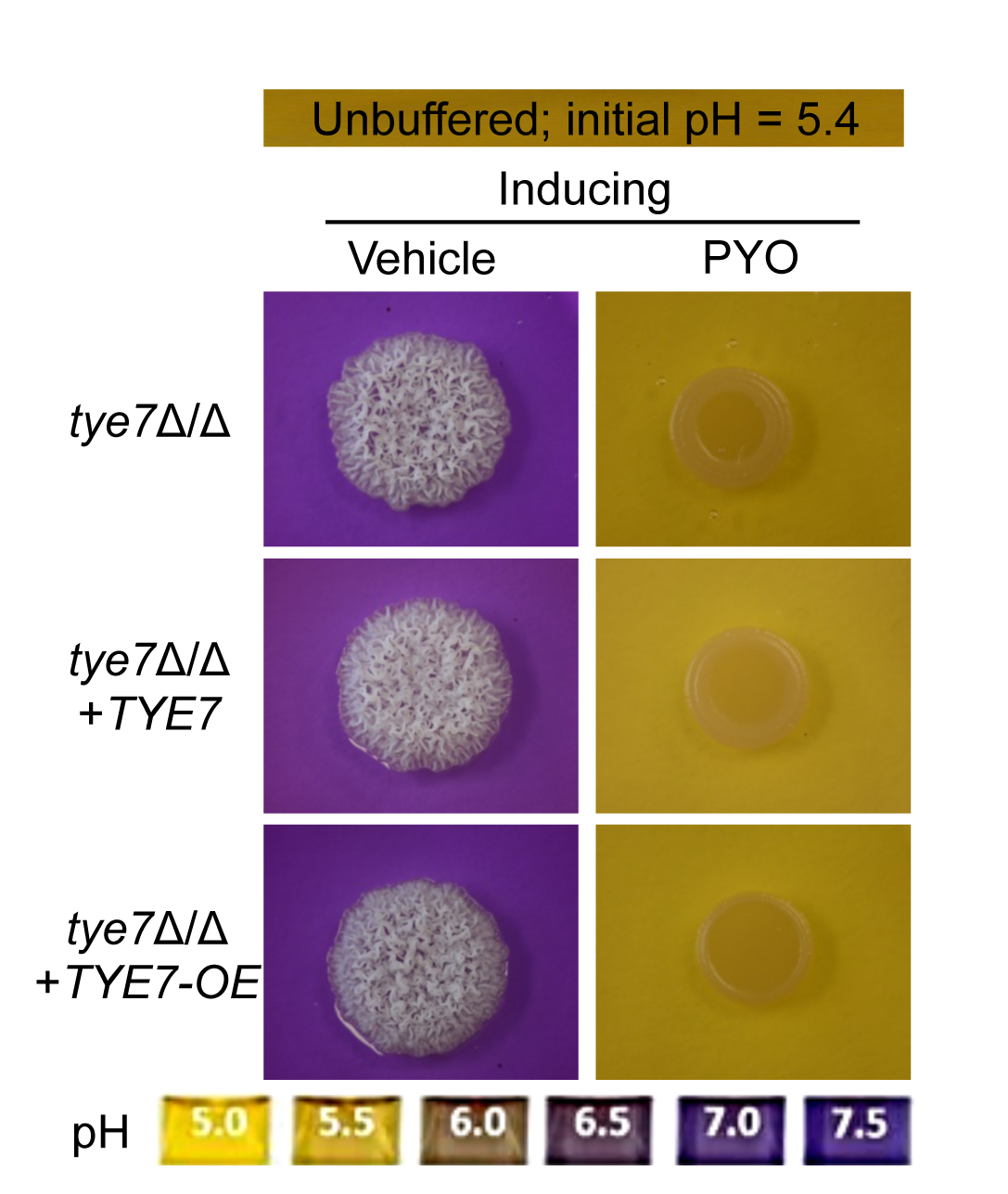

Supplement: Figure S12 — Altered expression of TYE7 does not influence wrinkling, alkalinization or sensitivity to PYO. Wrinkling, alkalinization and sensitivity to PYO were compared for tye7Δ/Δ, tye7Δ/Δ+TYE7 and tye7Δ/Δ+TYE7-OE. Colonies were grown on YNBAG10N with vehicle or 20 µM PYO at 37°C for 48 h and then imaged using a digital camera. (TIF) [file pgen.1004567.s012.tif]
