# Supplementary material for: Analysis of Candida albicans Mutants Defective in the Cdk8 Module of Mediator Reveal Links between Metabolism and Biofilm Formation
Source: PLoS Genet. 2014 Oct 2;10(10):e1004567. doi: 10.1371/journal.pgen.1004567 (PMC4183431; doi:10.1371/journal.pgen.1004567)
Supplement: Table S1 — Mutants with increased resistance to the inhibitory effect of PYO on wrinkling. (DOCX) [file pgen.1004567.s013.docx]

**Table S1** Mutants with increased resistance to the inhibitory effect of PYO on wrinkling.

| Name | ORF# | Description | Non-  inducing | Inducing | |
| --- | --- | --- | --- | --- | --- |
|  |  |  |  | Vehicle | PYO |
| SC5314 | N/A | Wild type | - | +++ | - |
| CAF2 | N/A | Wild type | - | +++ | - |
| Day286 | N/A | Wild type | - | +++ | - |
| *SSN3* (DH1740) | 19.794 | Putative RNA polymerase II holoenzyme cyclin-dependent protein kinase component catalytic subunit involved in transcriptional regulation | - | +++ | +++ |
| *SSN8*  (DH1741) | 19.7355 | Component of RNA polymerase II holoenzyme involved in transcriptional regulation | - | +++ | +++ |
| *HOF1* | 19.5664 | Protein required for cytokinesis | + | +++ | +++ |
| *MEC1* | 19.1283 | CCCP^1^ with a role in DNA integrity and repair | + | +++ | ++ |
| *CAS5* | 19.4670 | TF^2^ involved in cell wall damage response | + | +++ | + |
| *DUN1* | 19.4002 | SR^3^ protein kinase involved in DNA damage CCCP | + | +++ | + |
| *YBL053* | 19.4136 | Uncharacterized | + | +++ | + |
| *GIN4* | 19.663 | Autophosphorylated kinase with a role in pseudohyphal-hyphal switch and cytokinesis | + | +++ | + |
| *CHS7* | 19.2444 | Protein required for WT chitin synthase III activity | + | +++ | + |
| *ULP2* | 19.4353 | SUMO^4^ de-conjugation enzyme | + | +++ | + |
| *Unnamed* | 19.5848 | RING-type zinc finger protein | + | +++ | + |
| *SKY1* | 19.2436 | Putative SR protein kinase involved in regulating mRNA metabolism and cation homeostasis | + | +++ | +- |
| *KIS1* | 19.4084 | Snf1 complex scaffold protein | + | +++ | +- |

^1^*CCCP:* cell cycle checkpoint protein; ^2^TF: transcription factor; ^3^Serine-threonine; ^3^Small ubiquitin-like modifier
