# Supplementary material for: Analysis of Candida albicans Mutants Defective in the Cdk8 Module of Mediator Reveal Links between Metabolism and Biofilm Formation
Source: PLoS Genet. 2014 Oct 2;10(10):e1004567. doi: 10.1371/journal.pgen.1004567 (PMC4183431; doi:10.1371/journal.pgen.1004567)
Supplement: Table S3 — Absence of Ssn3 results in decreased expression of HSP12 and CTA1. (DOCX) [file pgen.1004567.s015.docx]

**Table S3** Absence of Ssn3 results in decreased expression of *HSP12* and *CTA1.*

|  |  | Transcript levels normalized to *PMA1* | | | | | |
| --- | --- | --- | --- | --- | --- | --- | --- |
|  |  | *HSP12* |  |  |  | *CTA1* |  |
| Strain | Average | SEM | P-value |  | Average | SEM | P-value |
| Wild type | 1.20 | 0.02 | n/a |  | 1.069 | 0.0175 | n/a |
| *ssn3*Δ/Δ | 0.47 | 0.09 | 0.008 |  | 0.294 | 0.0198 | 0.003 |
| *ssn3*Δ/Δ+*SSN3* | 1.38 | 0.15 | 0.371 |  | 0.495 | 0.0002 | 0.028 |
