# Supplementary material for: Analysis of Candida albicans Mutants Defective in the Cdk8 Module of Mediator Reveal Links between Metabolism and Biofilm Formation
Source: PLoS Genet. 2014 Oct 2;10(10):e1004567. doi: 10.1371/journal.pgen.1004567 (PMC4183431; doi:10.1371/journal.pgen.1004567)
Supplement: Table S4 — Absence of Ssn3 results in decreased levels of three of the twenty key amino acids. (DOCX) [file pgen.1004567.s016.docx]

**Table S4** Absence of Ssn3 results in decreased levels of three of the twenty key amino acids.

| **Metabolite** | **Δ/Δ^1^** | ***SSN3*** | **Δ/Δ** |
| --- | --- | --- | --- |
|  | **WT** | **WT** | ***SSN3*** |
| Leucine | **0.73** | 1.01 | **0.72** |
| Isoleucine | **0.79** | 1.12 | **0.70** |
| Phenylalanine | **0.76** | 0.98 | **0.78** |

^1^∆/∆ refers to the *ssn3*∆/∆ mutant. Bold font represents a statistically significant difference (p < 0.05).
