# Supplementary material for: Analysis of Candida albicans Mutants Defective in the Cdk8 Module of Mediator Reveal Links between Metabolism and Biofilm Formation
Source: PLoS Genet. 2014 Oct 2;10(10):e1004567. doi: 10.1371/journal.pgen.1004567 (PMC4183431; doi:10.1371/journal.pgen.1004567)
Supplement: Table S5 — Strains and plasmids used in this study. (DOCX) [file pgen.1004567.s017.docx]

**Table S5** Strains and plasmids used in this study.

| **Strain or Plasmid** | **Genotype** | **Source** | **Lab no.** |
| --- | --- | --- | --- |
| **Strains** | | | |
| SC5314 | Prototrophic clinical isolate | [[1](#_ENREF_1)] | DH35 |
| CAF2 | *URA3/ura3*::imm434 *IRO1/iro1*::imm434 | [[2](#_ENREF_2)] | DH331 |
| DAY286 | *ura3*∆*::λimm434/ura3*∆*::λimm434 ARG4:URA3:arg4::hisG/arg4::hisG his1::hisG/ his1::hisG* | [[3](#_ENREF_3)] | DH1740 |
| *ssn3*Δ/Δ | *ura3∆::λimm434/ura3∆::λimm434 arg4::hisG/arg4::hisG his1::hisG/his1::hisG ssn3*::*Tn7-UAU1/ssn3::Tn7-URA3* | [[3](#_ENREF_3)] | DH1741 |
| *ssn3*Δ/Δ*-pDDB78* | *ura3∆::λimm434/ura3∆::λimm434 arg4::hisG/arg4::hisG his1::hisG/his1::hisG*::*pHIS1* *ssn3*::*Tn7-UAU1/ssn3::Tn7-URA3* | This study | DH1969 |
| *ssn3*Δ/Δ*-pDDB78+SSN3* | *ura3∆::λimm434/ura3∆::λimm434 arg4::hisG/arg4::hisG his1::hisG/his1::hisG*::*pHIS1-SSN3* *ssn3*::*Tn7-UAU1/ssn3::Tn7-URA3* | This study | DH2073 |
| *ssn8*Δ/Δ | *ura3∆::λimm434/ura3∆::λimm434 arg4::hisG/arg4::hisG his1::hisG/his1::hisG ssn8*::*Tn7-UAU1/ssn8::Tn7-URA3* | [[3](#_ENREF_3)] | DH1742 |
| *ssn8*Δ/Δ*-pDDB78* | *ura3∆::λimm434/ura3∆::λimm434 arg4::hisG/arg4::hisG his1::hisG/his1::hisG*::*pHIS1* *ssn8*::*Tn7-UAU1/ssn8::Tn7-URA3* | This study | DH1970 |
| *ssn8*Δ/Δ*-pDDB78+SSN8* | *ura3∆::λimm434/ura3∆::λimm434 arg4::hisG/arg4::hisG his1::hisG/his1::hisG*::*pHIS1-SSN8* *ssn8*::*Tn7-UAU1/ssn8::Tn7-URA3* | This study | DH2076 |
| *ssn3*Δ/Δ *(SN982)*^1^ | *URA3/ura3::imm434 arg4/arg4::C.d.ARG4 his1/his1::ssn3::C.d. pHIS1 IRO1/iro1::imm434 leu2/leu2::ssn3::C.m.LEU2* | [[4](#_ENREF_4)] | DH2203 |
| *ssn3*Δ/Δ*+SSN3 (SN978)* | *URA3/ura3::imm434 arg4/arg4::SSN3::C.d.ARG4 his1/his1::ssn3::C.d.pHIS1 IRO1/iro1::imm434 leu2/leu2::ssn3::C.m.LEU2* | [[4](#_ENREF_4)] | DH2204 |
| *ssn3*Δ/Δ*+ssn3^D325A^ (SN987)* | *URA3/ura3::imm434 arg4/arg4::SSN3::C.d.ARG4-TDH3p-ssn3^D325A^-13xMyc-FLP-SAT1 his1/his1::ssn3::C.d.pHIS1 IRO1/iro1::imm434 leu2/leu2::ssn3::C.m.LEU2* | [[4](#_ENREF_4)] | DH2205 |
| *IRO1/iro1* | *MTLa/a arg4/arg4 leu2/leu2 his1/his1 URA3/ura3::imm434 IRO1/iro1::imm434* | [[5](#_ENREF_5)] | DH1889 |
| *srb8*Δ/Δ | *MTLa/a arg4/arg4 leu2/leu2 his1/his1 URA3/ura3::imm434 IRO1/iro1::imm434 srb8Δ::HIS1/srb8Δ::LEU2* | [[6](#_ENREF_6)] | DH1891 |
| *srb8*Δ/Δ+*SRB8* | *As srb8*Δ/Δ*, but SRB8+ARG4* | [[6](#_ENREF_6)] | DH1935 |
| *srb9*Δ/Δ | *ura3∆::λimm434/ ura3∆::λimm434 arg4::hisG/arg4::hisG his1::hisG/his1::hisG*::*pHIS1 srb9*::*ARG4/srb9::URA3* | [[7](#_ENREF_7)] | DH1971 |
| *srb9*Δ/Δ*+SRB9* | *ura3∆::λimm434/ ura3∆::λimm434 arg4::hisG/arg4::hisG his1::hisG/his1::hisG*::*pHIS1-SRB9 srb9*::*ARG4/srb9::URA3* | [[7](#_ENREF_7)] | DH1972 |
| *SSN3-3HA/SSN3-3HA* | *ura3*∆*::λimm434/ura3∆::λimm434 his1∆::hisG/his1∆::hisG arg4∆::hisG/arg4∆::hisG SSN3::SSN3-3XHA-HIS1/SSN3::SSN3-3XHA-SAT1* | This study | DH2209 |
| *SSN8-3HA/SSN8-3HA* | *ura3∆::λimm434/ura3∆::λimm434 his1∆::hisG/his1∆::hisG arg4∆::hisG/arg4∆::hisG SSN8::SSN8-3XHA-ARG4/SSN8::SSN8-3XHA-HIS1* | This study | DH2216 |
| *med20*Δ/Δ | *ura3∆::λimm434/ ura3∆::λimm434 arg4::hisG/arg4::hisG his1::hisG/his1::hisG*::*pHIS1 srb9*::*ARG4/srb9::URA3* | [[7](#_ENREF_7)] | DH1973 |
| *med1*Δ/Δ | *MTLa/a arg4/arg4 leu2/leu2 his1/his1 URA3/ura3::imm434 IRO1/iro1::imm434 med1Δ::HIS1/med1Δ::LEU2* | [[6](#_ENREF_6)] | DH1924 |
| *med5*Δ/Δ | *MTLa/a arg4/arg4 leu2/leu2 his1/his1 URA3/ura3::imm434 IRO1/iro1::imm434 med5Δ::HIS1/med5Δ::LEU2* | [[6](#_ENREF_6)] | DH1919 |
| *med9*Δ/Δ | *MTLa/a arg4/arg4 leu2/leu2 his1/his1 URA3/ura3::imm434 IRO1/iro1::imm434 med9Δ::HIS1/med9Δ::LEU2* | [[6](#_ENREF_6)] | DH1927 |
| *med16*Δ/Δ | *MTLa/a arg4/arg4 leu2/leu2 his1/his1 URA3/ura3::imm434 IRO1/iro1::imm434 med16Δ::HIS1/med16Δ::LEU2* | [[6](#_ENREF_6)] | DH1921 |
| *med3*Δ/Δ | *MTLa/a arg4/arg4 leu2/leu2 his1/his1 URA3/ura3::imm434 IRO1/iro1::imm434 med3Δ::HIS1/med3Δ::LEU2* | [[6](#_ENREF_6)] | DH1933 |
| *tye7*Δ/Δ  (JCP200) | *ura3∆::λimm434/ ura3∆::λimm434-URA3-IRO1 arg4::hisG/arg4::hisG his1::hisG/his1::hisG leu2::hisG::CdHIS1/ leu2::hisG::CmLEU2 TYE7::* *AgTEF1p+NAT1+AgTEF1UTR+TDH3p* | [[8](#_ENREF_8)] | DH2124 |
| *tye7*Δ/Δ-*TYE7*  *(*JCP196) | *ura3∆::λimm434/ ura3∆::λimm434-URA3-IRO1 arg4::hisG/arg4::hisG his1::hisG/his1::hisG leu2::hisG::/ leu2::hisG tye7::CdHIS1/tye7::CmLEU2 rsp10::TYE7-SAT1/RSP10* | [[8](#_ENREF_8)] | DH2123 |
| *tye7*Δ/Δ-*TYE7-OE*  *(*JCP272) | *ura3∆::λimm434/ ura3∆::λimm434-URA3-IRO1 arg4::hisG/arg4::hisG his1::hisG/his1::hisG leu2::hisG::CdHIS1/ leu2::hisG::CmLEU2 TYE7::* *AgTEF1p+NAT1+AgTEF1UTR+TDH3p+TYE7/TYE7* | [[8](#_ENREF_8)] | DH2122 |
| **Plasmids** |  |  |  |
| *pDDB78* | *HIS* integrating plasmid | [[3](#_ENREF_3)] | DH1745 |
| *pFA-3XHA-HIS1* |  | [[9](#_ENREF_9)] | DH2233 |
| *pFA-3HA-SAT1* |  | [[9](#_ENREF_9)] | DH2234 |
| *pFA-3HA-ARG4* |  | [[9](#_ENREF_9)] | DH2235 |

*^1^C.d. ARG4* denotes *Candida dubliniensis ARG4, C.d. HIS1* denotes *Candida dubliniensis HIS1,* and *C.m. LEU2* denotes *Candida maltosa* *LEU2*.

References

1. Gillum AM, Tsay EY, Kirsch DR (1984) Isolation of the *Candida albicans* gene for orotidine-5'-phosphate decarboxylase by complementation of *S. cerevisiae* ura3 and *E. coli* *pyrF* mutations. Mol Gen Genet 198: 179-182.

2. Gow NA, Brown AJ, Odds FC (2002) Fungal morphogenesis and host invasion. Curr Opin Microbiol 5: 366-371.

3. Blankenship JR, Fanning S, Hamaker JJ, Mitchell AP (2010) An extensive circuitry for cell wall regulation in *Candida albicans*. PLoS Pathog 6: e1000752.

4. Chen C, Noble SM (2012) Post-transcriptional regulation of the Sef1 transcription factor controls the virulence of *Candida albicans* in its mammalian host. PLoS Pathog 8: e1002956.

5. Noble SM, Johnson AD (2005) Strains and strategies for large-scale gene deletion studies of the diploid human fungal pathogen *Candida albicans*. Eukaryot Cell 4: 298-309.

6. Zhang A, Liu Z, Myers LC (2013) Differential regulation of white-opaque switching by individual subunits of *Candida albicans* mediator. Eukaryot Cell 12: 1293-1304.

7. Uwamahoro N, Qu Y, Jelicic B, Lo TL, Beaurepaire C, et al. (2012) The functions of Mediator in *Candida albicans* support a role in shaping species-specific gene expression. PLoS Genet 8: e1002613.

8. Perez JC, Kumamoto CA, Johnson AD (2013) *Candida albican*s commensalism and pathogenicity are intertwined traits directed by a tightly knit transcriptional regulatory circuit. PLoS Biol 11: e1001510.

9. Zhang A, Petrov KO, Hyun ER, Liu Z, Gerber SA, et al. (2012) The Tlo proteins are stoichiometric components of *Candida albicans* mediator anchored via the Med3 subunit. Eukaryot Cell 11: 874-884.
