# Supplementary material for: Analysis of Candida albicans Mutants Defective in the Cdk8 Module of Mediator Reveal Links between Metabolism and Biofilm Formation
Source: PLoS Genet. 2014 Oct 2;10(10):e1004567. doi: 10.1371/journal.pgen.1004567 (PMC4183431; doi:10.1371/journal.pgen.1004567)
Supplement: Table S6 — Primers used in this study. (DOCX) [file pgen.1004567.s018.docx]

**Table S6** Primers used in this study.

| Target | Primer ID | Sequence |  |
| --- | --- | --- | --- |
| *Complementation* | | | |
| *SSN3* orf19.794 | SSN3c2-F | Forward: 5’-TTC ACA CAG GAA ACA GCT ATG ACC ATG ATT ACG CCA AGC TCT TTA CAA CGA GAA ATC ATT TC-3’ | |
|  | SSN3c2-R | Reverse: 5’-TCG ACC ATA TGG GAG AGC TCC CAA CGC GTT GGA TGC ATA GAA CTT GTA GTT GTT GCT TTA TG-3’ | |
| *SSN8* orf19.7355 | SSN8c2-F | Forward: 5’-TTC ACA CAG GAA ACA GCT ATG ACC ATG ATT ACG CCA AGC TTT GCC AAG CTG TCT AGA AAA TG-3’ | |
|  | SSN8c2-R | Reverse: 5’-TCG ACC ATA TGG GAG AGC TCC CAA CGC GTT GGA TGC ATA GCT TTT TCG CAT TTC CCA CAC AC-3’ | |
| *HA-Tagging* |  |  | |
|  | SSN3tag sense | Forward: 5’-CTG CAT GGT GCA AAC AGT ACA TAT AAG CGA AGT GGT ATT GAT GAT TTA CCT GGT GGA ATT AGA AAG AAA CGT GGG ggt cga cgg atc ccc-3’ | |
|  | SSN3tag anti | Reverse: 5’-CAC CAC CAC CAT CAA AAT TCA ATT CAT TTA CGA TCA TTA AAA CAC TAT ACA AAT AAA AAT GAC ATC TAT ATC TAT tcg atg aat tcg agc-3’ | |
|  | SSN8tag sense | Forward: 5’-ATG TAT GTA TTA TGG AAT CGA TAT AAT GAA CAA GGG GTT AAA AAG GCA TTA CAA GTG ATG TTA CTC AAT CGG ATT ggt cga cgg atc ccc-3’ | |
|  | SSN8tag anti | Reverse: 5’-ATA TTT ATT TCC AGG AAA CTT CTT CTT CTT CTG AGT CTG GAA ATT GTT CTT TTT TTG TTA ATG TTA AAC TTT tcg atg aat tcg agc tcg-3’ | |
| *RT-PCR* |  |  | |
| *CDC19 orf19.3575* | CDC19F | Forward: 5′-TCT GGT GAA ACC GCT AAA GG-3’ | |
|  | CDC19R | Reverse: 5’-GTA GGC AAT GGC CTT TTC AG-3’ | |
| *ENO1 orf19.395* | ENO1F | Forward: 5′-AAA TCC ACG CCA GAT ACG TC-3’ | |
|  | ENO1R | Reverse: 5’-CCT TTG TCG GTG GTG AAA TC-3’ | |
| *FBA1 orf19.4618* | FBA1F | Forward: 5′-TGG TGG TGA AGA AGA TGG TG-3’ | |
|  | FBA1R | Reverse: 5’-ACC GTG GAC GTT ACC AAA AG-3’ | |
| *GPM1 orf19.903* | GPM1F | Forward: 5′-CCA CCA CCA AAG ATT GAT CC-3’ | |
|  | GPM1R | Reverse: 5’-TGG ATC AAC GTC AGC GTA TC-3’ | |
| *GPM2 orf19.1067* | GPM2F | Forward: 5′-CAT GGA TTG CCA CCC TTA ATA G-3’ | |
|  | GPM2R | Reverse: 5’-CGG AAT CAA TCT GTC CAT CAC-3’ | |
| *HXK2 orf19.542* | HXK2F | Forward: 5′-CAA TGG CCA TCA ACT GTG AG-3’ | |
|  | HXK2R | Reverse: 5’-TTG TTG ACC TGG TCT TGG TG-3’ | |
| *PFK1 orf19.3967* | PFK1F | Forward: 5′-GGT TTA AAT GCT GCC ACT CG-3’ | |
|  | PFK1R | Reverse: 5’-CCA TCC TTC AAC ATC CAT CC-3’ | |
| *PFK2 orf19.6540* | PFK2F | Forward: 5′-TTC AGG TGG GGA TTC TCA AG-3’ | |
|  | PFK2R | Reverse: 5’-ACC ACC TTT GAC CAA ACC AG-3’ | |
| *PGI1 orf19.395* | PGI1F | Forward: 5′-CCA CAT CAA CAC AAC CGA AG-3’ | |
|  | PGI1R | Reverse: 5’-TGA GCG GTA TCT TTC CCA TC-3’ | |
| *PGK1 orf19.3888* | PGK1F | Forward: 5′-AAC GAT GCC TTT GGT ACT GC-3’ | |
|  | PGK1R | Reverse: 5’-GGG TTT TCC AAA GCC TTA GC-3’ | |
| *TPI1 orf19.6745* | TPI1F | Forward: 5′-TTT GTC CAC CAG CCC TTT AC-3’ | |
|  | TPI1R | Reverse: 5’-TTC ACC AGT GAA AGC ACC AC-3’ | |
| *HGT2* orf19.11152 | HGT2F | Forward: 5′- ACT GGT ATG AAC GTT ATG ATG-3’ | |
|  | HGT2R | Reverse: 5′-ACT GAG TCG TTA CCA CTA TCT-3’ | |
| *HXT5* orf19.4384 | HXT5F | Forward: 5′- TGG TTT GAG TTG GTC ACC ATC-3’ | |
|  | HXT5R | Reverse: 5′- GAA TGG TAC CAA TAA GCA ACG AC-3’ | |
| *CTA1* orf19.6229 | CTA1_RTF | Forward: 5′-CCA GAT TAC GCC CAA GAA GAT-3′ | |
|  | CTA1_RTR | Reverse: 5′-AAC AGT GTG GGC TGG AGA GAA-3′ | |
| *HSP12* orf19.3160 | HSP12F | Forward: 5′- TGT TGG CTC AAA TGT TCC AG-3′ | |
|  | HSP12R | Reverse: 5′- TTC AGC AGC CTT TCC AAT TT-3′ | |
| *PMA1* *orf19.5383* | PMA1 F | Forward: 5’-TTG CTT ATG ATA ATG CTC CAT ACG A-3’ | |
|  | PMA1 R | Reverse: 5’-TAC CCC ACA ATC TTG GCA AGT-3’ | |
